# Increasing spin crossover cooperativity in 2D Hofmann-type materials with guest molecule removal†
†Electronic supplementary information (ESI) available: Experimental information, and synthesis and characterisation of materials (PDF). CCDC 1519487–1519489. For ESI and crystallographic data in CIF or other electronic format see DOI: 10.1039/c8sc01040d


**DOI:** 10.1039/c8sc01040d

**Published:** 2018-05-29

**Authors:** Katrina A. Zenere, Samuel G. Duyker, Elzbieta Trzop, Eric Collet, Bun Chan, Patrick W. Doheny, Cameron J. Kepert, Suzanne M. Neville

**Affiliations:** a School of Chemistry , The University of Sydney , Sydney , New South Wales 2006 , Australia . Email: cameron.kepert@sydney.edu.au; b Univ Rennes , CNRS , IPR (Institut de Physique de Rennes) – UMR 6251 , F-35000 Rennes , France; c Graduate School of Engineering , Nagasaki University , 1-14 Bunkyo-machi , Nagasaki-shi Nagasaki 852-8521 , Japan; d School of Chemistry , The University of New South Wales , Kensington , New South Wales 2052 , Australia . Email: s.neville@unsw.edu.au

## Abstract

Molecule-based spin state switching materials that display ambient temperature transitions with accompanying wide thermal hysteresis offer an opportunity for electronic switching, data storage, and optical technologies but are rare in existence. Here, we present the first 2D Hofmann-type materials to exhibit the elusive combination of ambient temperature spin crossover with wide thermal hysteresis (Δ*T* = 50 and 65 K). Combined structural, magnetic, spectroscopic, and theoretical analyses show that the highly cooperative transition behaviours of these layered materials arise due to strong host–host interactions in their interdigitated lattices, which optimises long-range communication pathways. With the presence of water molecules in the interlayer pore space in the hydrated phases, competing host–host and host–guest interactions occur, whilst water removal dramatically increases the framework cooperativity, thus affording systematic insight into the structural features that favour optimal spin crossover properties.

## Abstract 2

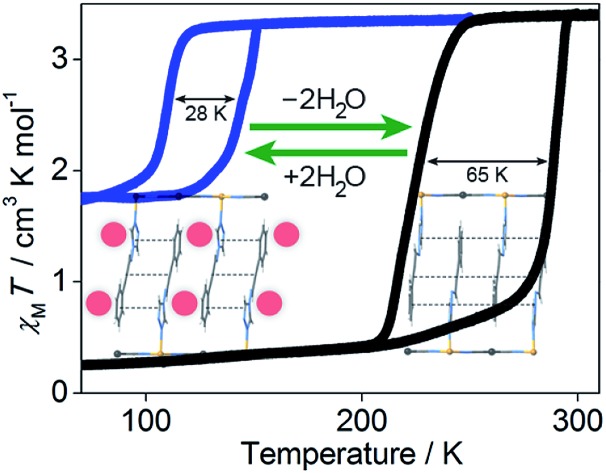
Ambient temperature spin crossover with wide hysteresis has been achieved in 2D Hofmann-type materials, where removal of guest molecules optimises ligand–ligand interactions, resulting in increased cooperativity.

## Introduction

Extensive studies on spin crossover (SCO) compounds have been conducted over the past few decades as they are prototypical examples of molecular systems exhibiting electronic bistability.^1^–^3^ Among these, Fe(ii) materials show a particularly diverse array of SCO behaviours, including gradual, abrupt, hysteretic, and multi-step spin transitions. The reversible switching between a high spin (HS, *S* = 2) and low spin (LS, *S* = 0) state, induced by an external stimulus (such as temperature, pressure, or light), may be accompanied by hysteresis when long-range elastic interactions mediate spin state cooperativity.^4^ This molecular bistability is a significant feature for the development of molecule-based electronic devices for switching, sensing, and information storage applications.^2^,^5^–^8^

The utility of SCO materials for many practical applications relies on the presence of ambient working conditions, abrupt transitions (preferably with a large thermal hysteresis, Δ*T* > 50 K), and cyclability without fatigue.^2^,^4^–^7^,^9^,^10^ Although many SCO materials are known that either exhibit ambient temperature spin transitions or wide hysteresis,^11^–^17^ examples that exhibit the combination of these two features is still relatively uncommon.^18^–^25^ Examples of hysteretic systems include both discrete and polymeric metallosupramolecular materials with a common (but not prescriptive) feature being the presence of prominent intermolecular contacts (such as hydrogen bonding and aromatic interactions) and/or direct coordination bonds between SCO sites which facilitate spin state cooperativity; notably, numerous non-hysteretic SCO materials also contain such communication pathways, indicating considerable complexity in the structure–property relationships at play.^1^–^3^

Compared to discrete SCO complexes, which predominately rely on intermolecular interactions (such as hydrogen bonding, van der Waals forces, and aromatic stacking) to influence spin state cooperativity, polymeric network systems generally exhibit stronger cooperativity between the SCO sites which are directly linked through covalent bonds to create a rigid lattice.^26^ Characteristic of porous framework materials is that guest molecules have a significant effect on the SCO behaviour, thus these framework materials provide a unique foundation for manipulating SCO features by exploiting host–guest chemistry. Numerous guest-dependent SCO studies have been reported which demonstrate the unique synergistic interactions between the host system and guest molecules, which often influences the cooperativity.^27^ The effect of guest molecules on SCO behaviour and lattice cooperativity is clearly demonstrated in [Fe(bpbd)_2_(NCS)_2_]·*n*(guest) (bpbd = 2,3-bis(4′-pyridyl)-2,3-butanediol; guest = acetonitrile, acetone, methanol, ethanol, 1-propanol), which shows a general trend between transition temperature and guest, and where thermal hysteresis (which is indicative of strong cooperativity) appears for the ethanol and methanol species as a result of strong host–guest interactions.^28^ More extreme variations in SCO behaviour with guest manipulation are observed in the 3D Hofmann-type framework [Fe(pz)Ni(CN)_4_]·*n*(guest) (pz = pyrazine; guest = water, methanol, ethanol, acetone, acetonitrile, toluene), where a dynamic interplay between two phenomena occur, namely SCO behaviour being induced by guest-exchange and the emergence of SCO-induced host–guest properties, such that this system exhibits a guest-dependent memory effect.^29^ Moreover, unique guest-programmable multi-step SCO has been observed in the 2D Hofmann-type framework [Fe(bztrz)_2_Pd(CN)_4_]·*n*(guest) (bz = (*E*)-1-phenyl-*N*-(1,2,4-triazol-4-yl)methanimine), which encompasses one-, two-, three-step SCO behaviour, and SCO-deactivation within the one material depending on the guest contents, thus displaying the broadest range of spin transition character reported to date as a result of competing ferro- and antiferro-elastic interactions in the frustrated lattice.^30^

**Fig. 1:**
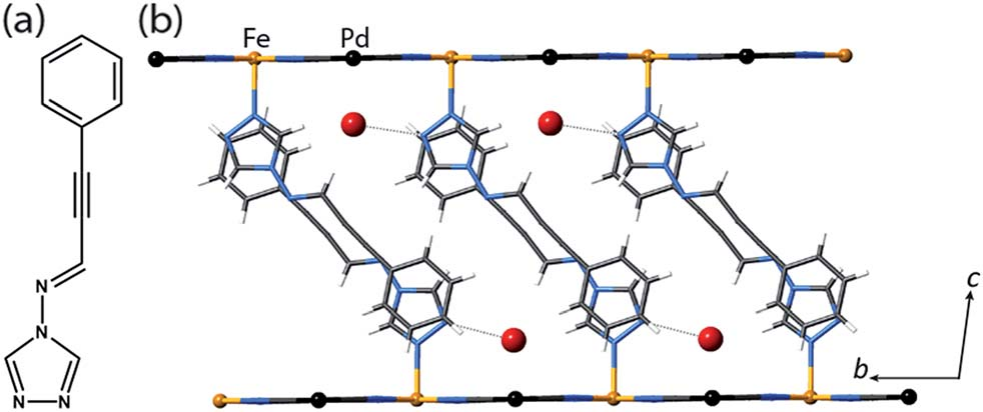
(a) proptrz = (*E*)-3-phenyl-*N*-(4*H*-1,2,4-triazol-4-yl)prop-2-yn-1-imine. (b) Single crystal structure of two neighbouring layers of **1(Pd)**·2H_2_O at 200 K (viewed in the *bc*-plane) highlighting host–guest interactions, ligand interdigitation, and the octahedral Fe(ii) coordination environment. (Water guest molecules are shown as large red spheres, metal ions as small spheres, and the rest of the framework as sticks.)

Encouraged by our recent findings that functionalised 1,2,4-triazole ligands, as a result of their asymmetric binding mode, are optimal for probing and modulating elastic interactions in flexible 2D Hofmann-type materials,^30^–^33^ we were motivated to explore the incorporation of a novel ligand (proptrz = (*E*)-3-phenyl-*N*-(4*H*-1,2,4-triazol-4-yl)prop-2-yn-1-imine; Fig. 1(a)) with strategically included hydrogen bonding and aromatic interaction sites to integrate an array of spin state communication pathways through host–host and host–guest interactions. Through this approach we have generated the first examples of 2D framework materials that exhibit the elusive combination of ambient temperature SCO and large hysteresis, as facilitated by the strategic integration of intra- and intermolecular interactions within a flexible 2D lattice. Of additional importance to this study, we find that cooperativity is less pronounced in the hydrated phases due to the hindrance of spin state communication pathways by water guest molecules in the pores, compared to the dehydrated phases in which cooperativity is enhanced due to the elimination of elastic lattice frustration from the guests and competing host–guest interactions.

## Results and discussion

### Framework synthesis and structure

Yellow crystals of the target materials, [Fe(proptrz)_2_M(CN)_4_]·2H_2_O (M = Pd or Pt; **1(Pd)**·2H_2_O, **1(Pt)**·2H_2_O), were produced by slow diffusion of Fe(ClO_4_)_2_·*x*H_2_O, K_2_[M(CN_4_)] (M = Pd or Pt), and proptrz (Fig. 1(a)) in a EtOH : H_2_O (50 : 50%) solution (see ESI†).

**Fig. 2:**
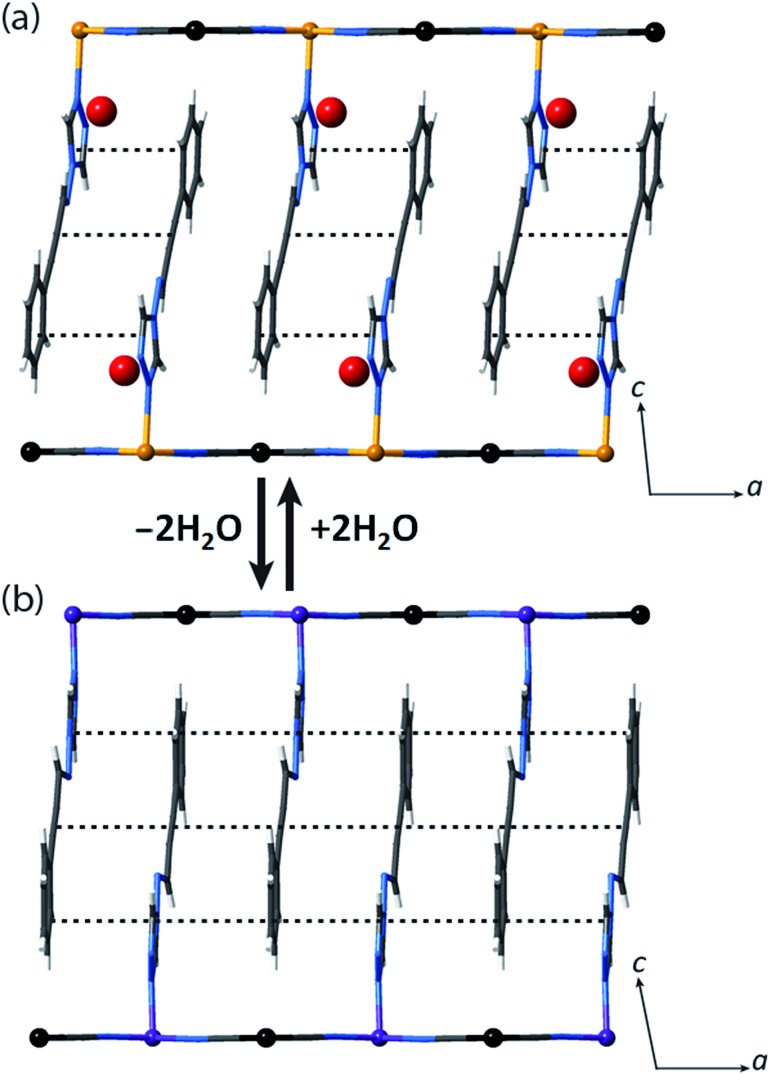
(a) Single crystal structure of **1(Pd)**·2H_2_O at 200 K (viewed in the *ac*-plane), showing the aromatic interactions between pairs of ligands. (b) Illustration of the likely structure of **1(Pd)** (viewed in the *ac*-plane) obtained from constrained Rietveld refinement of the LS structure at 150 K, which suggests that with guest water removal, optimisation of the ligand–ligand interactions occurs towards 1D chains.

**Fig. 3:**
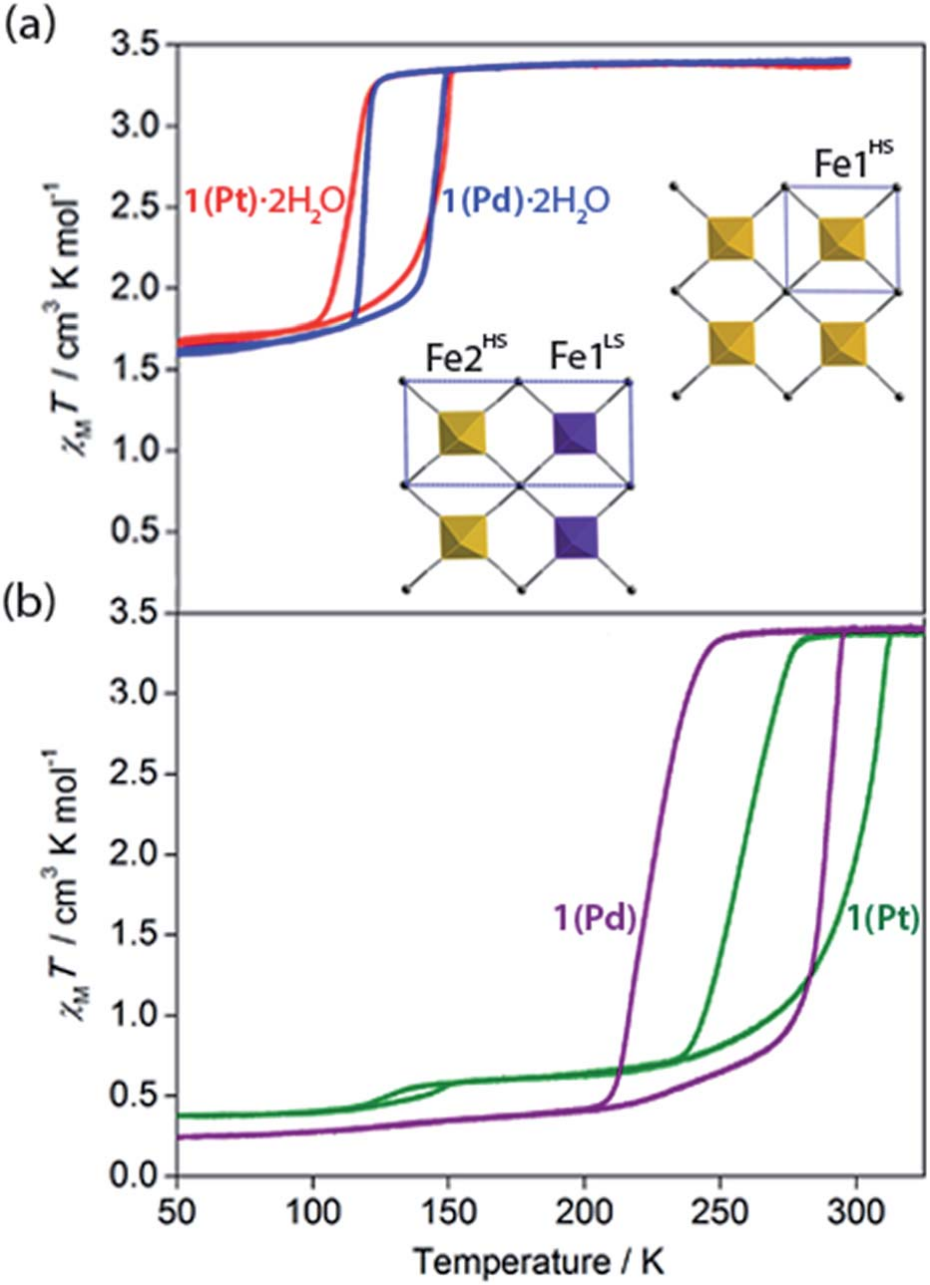
Variable temperature magnetic susceptibility of (a) **1(Pd)**·2H_2_O (blue) and **1(Pt)**·2H_2_O (red), and (b) **1(Pd)** (purple) and **1(Pt)** (green). Inset: single crystal structure of **1(Pd)**·2H_2_O (viewed in the *ab*-plane) at 110 K (left) and 200 K (right); HS (yellow) and LS (purple) sites are indicated; unit cell of each is indicated in blue.

Single crystal X-ray diffraction analyses of **1(Pd)**·2H_2_O and **1(Pt)**·2H_2_O at 200 K reveal isostructural 2D Hofmann-type topologies consisting of bimetallic 2D layers of Fe(ii) sites linked *via* [M(CN)_4_]^2–^ anions (M = Pd or Pt). Two monodentate proptrz ligands bind axially to each Fe(ii) site, completing the octahedral coordination environment and acting as spacers between adjacent 2D layers; these ligands interdigitate with near-complete ‘head-to-tail’ overlap (Fig. 1(b)) and, rather than stacking uniformly, orient in a dimerised fashion (Fig. 2(a)). Within the host lattice are interlayer spaces that accommodate two water molecules per Fe(ii) site. These guest molecules participate in hydrogen bonding interactions with the non-coordinated nitrogen atoms of the 1,2,4-triazole groups (Fig. 1(b), Table S2†), and are associated with (and potentially strongly influence) the pairing of the proptrz ligands along the *a*-direction (Fig. 2(a)); associated with this arrangement, the proptrz ligands show a subtle torsional twist of the triazole ring (Fig. S3(b)†). Structural analyses of **1(Pd)**·2H_2_O and **1(Pt)**·2H_2_O at 200 K reveal a single crystallographic Fe(ii) site (Fe1) with characteristic HS Fe–N bond lengths (*d*_Fe1–N_ = 2.16 Å for both **1(Pd)**·2H_2_O and **1(Pt)**·2H_2_O; Fig. 3(a) inset, Fig. S1, S6, Table S2†).

### Spin crossover properties of the hydrated phases

**Fig. 4:**
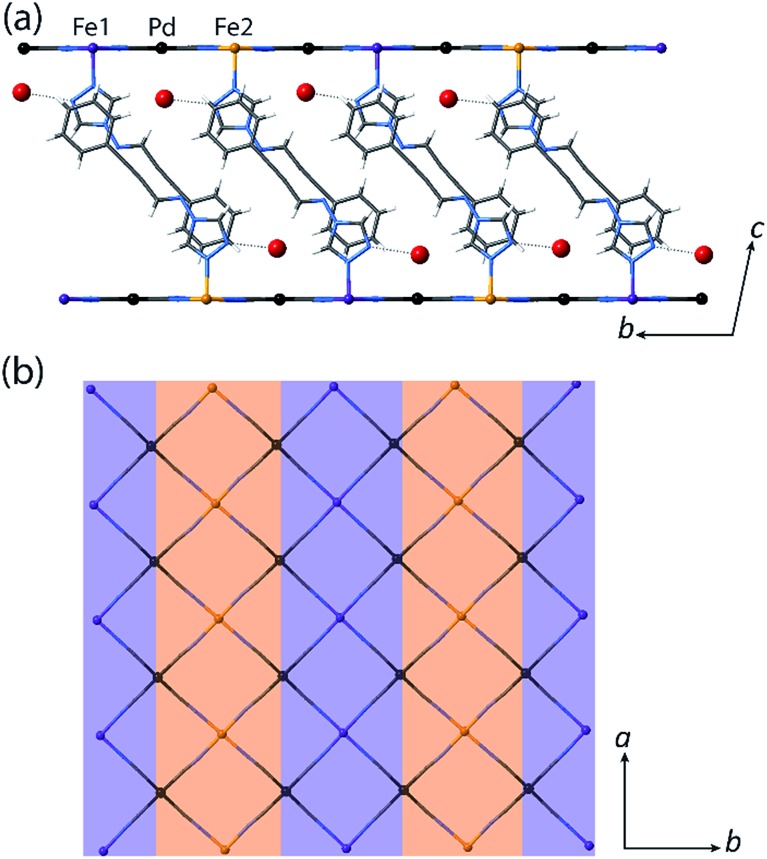
Single crystal structure of **1(Pd)**·2H_2_O at 110 K highlighting the ordering of the HS (orange) and LS (purple) sites in the (a) *bc*-plane and (b) *ab*-plane. Ligands and water molecules are omitted in (b) for clarity.

Variable temperature magnetic susceptibility measurements on both **1(Pd)**·2H_2_O and **1(Pt)**·2H_2_O reveal a one-step ‘half’ spin state transition of Fe(ii) sites with a moderate thermal hysteresis (**1(Pd)**·2H_2_O: *T*↓1/2 = 119 K, *T*↑1/2 = 147 K, Δ*T* = 28 K; **1(Pt)**·2H_2_O: *T*↓1/2 = 116 K, *T*↑1/2 = 149 K, Δ*T* = 33 K; measured in sweep mode at 2 K min^–1^; Fig. 3(a)). The magnetic susceptibility (*χ*_M_*T*) values at the high and low temperature plateau regions correspond to a transition from 100% HS to 50 : 50% HS : LS Fe(ii) sites, respectively. Structural analysis conducted at the low temperature plateau region (*T* = 110 K) reveals a doubling of the unit cell *b*-parameter, along with a conversion from one Fe(ii) site (Fe1 at 200 K) to two crystallographically distinct sites (Fe1 and Fe2 at 110 K) over the SCO process. This unit cell transformation yields 3D long-range ordering of LS and HS sites at low temperature (*d*_Fe1–N_ = 1.97 Å, *d*_Fe2–N_ = 2.14 Å for **1(Pd)**·2H_2_O; Fig. 3(a) inset, Fig. 4, S2†). The spin state ordering is commensurate with the 50 : 50% mixture of HS and LS sites determined magnetically at this temperature. Distinct HS and LS state ordering is present within each layer in the form of alternating HS and LS 1D stripes along the *a*-axis (Fig. 3(a) inset, Fig. 4, S5†). This spatial modulation translates to a 3D spin state configuration where communication between different Hofmann layers is facilitated through the interlayer interactions.

Several recent examples of Hofmann-type, Hofmann-like, and metallocyanide-based materials highlight the ability of such systems to accommodate fractional HS and LS sites through geometric competition between SCO sites.^30^,^31^,^33^–^36^ Indeed, recent theoretical and experimental studies show that the presence of competing ferro- and antiferro-elastic interactions drive such SCO-induced lattice distortions, in turn distinguishing fractional HS and LS sites representative of multi-step SCO.^37^,^38^ The array of ligand–ligand and ligand–guest interactions that are present in **1(Pd)**·2H_2_O and **1(Pt)**·2H_2_O provides a sufficient breadth of competitive interactions to afford such lattice frustration. This lattice frustration is evident in the evolution of competing distortion around the SCO sites within the Hofmann layers. Here, the single crystallographic Fe(ii) site of **1(Pd)**·2H_2_O in the HS state (octahedral distortion parameter,^39^*Σ*(Fe1_HS_) = 15.7° at 200 K) converts to a more regular LS site (*Σ*(Fe1_LS_) = 12.5° at 110 K) over the SCO process at the expense of greatly increasing the distortion around the neighbouring residual HS site (*Σ*(Fe2_HS_) = 28.2° at 110 K) in the 50 : 50% HS : LS lattice. The antiferro-elastic coupling breaks the symmetry and stabilises a thus spatial sequence of adjacent LS (Fe1) and HS (Fe2) sites, preventing full conversion to the LS lattice, even with further cooling. The steric bulk of the water molecules are a likely contributing factor in stabilising Fe2 in the HS state through internal pressure effects, alongside the geometric antagonism generated by the presence of competing host–host and host–guest interactions.

### Properties of dehydration

With these effects in mind, efforts were made to probe the structural and magnetic consequences of guest water removal from the host lattice. Thermogravimetric analyses show that the guest water molecules are readily desorbed with heating above 370 K to produce the guest-free phases, **1(Pd)** and **1(Pt)** (Fig. S33†). Magnetic susceptibility measurements on **1(Pd)** and **1(Pt)** reveal abrupt and complete one-step spin transitions with substantially increased transition temperatures and hysteresis loop widths (*i.e.*, cooperativity) compared to the hydrated phases (**1(Pd)**: *T*↓1/2 = 220 K, *T*↑1/2 = 285 K, Δ*T* = 65 K; **1(Pt)**: *T*↓1/2 = 257 K, *T*↑1/2 = 307 K, Δ*T* = 50 K; measured in sweep mode at 2 K min^–1^; Fig. 3(b)). (Note that the minor hysteretic feature observed at *ca.* 130 K in the magnetic susceptibility data of **1(Pt)** (Fig. 3(b)) corresponds to a partial (<5%) retention of the hydrated phase; this arose despite efforts to completely desolvate the sample, possibly due to pore collapse at the surface of the crystals attributed to this being a 2D Hofmann-type material with interdigitating ligands. This gives an indication of the highly hydrophilic nature of these frameworks, potentially associated with the uncoordinated nitrogen atom of the triazole part of the proptrz ligand.) Notably, both of these dehydrated materials show ambient temperature spin state switching with thermal hysteresis loops that are by far the widest reported for any 2D framework material and which are commensurate with the widest achieved in 3D Hofmann-type materials.^13^,^20^,^21^ Interestingly, despite the hydrated Pd and Pt phases showing near identical SCO behaviours with only marginal differences in their transition temperatures, their respective dehydrated phases show a clear distinction in both transition temperature and hysteresis loop width; the reason for this is unknown but may relate to variation in sample quality since **1(Pt)** shows significantly reduced crystallinity compared to **1(Pd)** (Fig. S17 and S21†).^40^

Overall, guest removal from **1(M)**·2H_2_O results in a change from an incomplete to a complete spin transition, alongside a significant increase in both the transition temperature (*T*_1/2_ increase of *ca.* 150 K) and hysteresis loop width (Δ*T* increase of *ca.* 30 K). While elastic frustration through geometric competition within the Hofmann layers of **1(M)**·2H_2_O results in the ‘striped’ 50 : 50% HS : LS state, no such effect is apparent in **1(M)** (as it is a complete HS to LS transition), confirming that guest molecules can play an important role in mediating elastic frustration.^38^ Collectively, these considerable differences in SCO behaviour between the hydrated and dehydrated phases suggest that commencement of the spin transition (either upon cooling or warming) in **1(M)** involves a smaller local energy penalty (which would otherwise restrict SCO) at the Fe(ii) sites than for **1(M)**·2H_2_O; the lessening of this penalty in **1(M)**, and the associated energy barrier for cooperative spin conversion, may relate in part to: (1) the difference between complete and partial SCO in **1(M)** and **1(M)**·2H_2_O, respectively, (2) a change in competition between antiferro-elastic and ferro-elastic effects with dehydration, and (3) an increase in lattice cooperativity with the removal of guest water molecules.

**Fig. 5:**
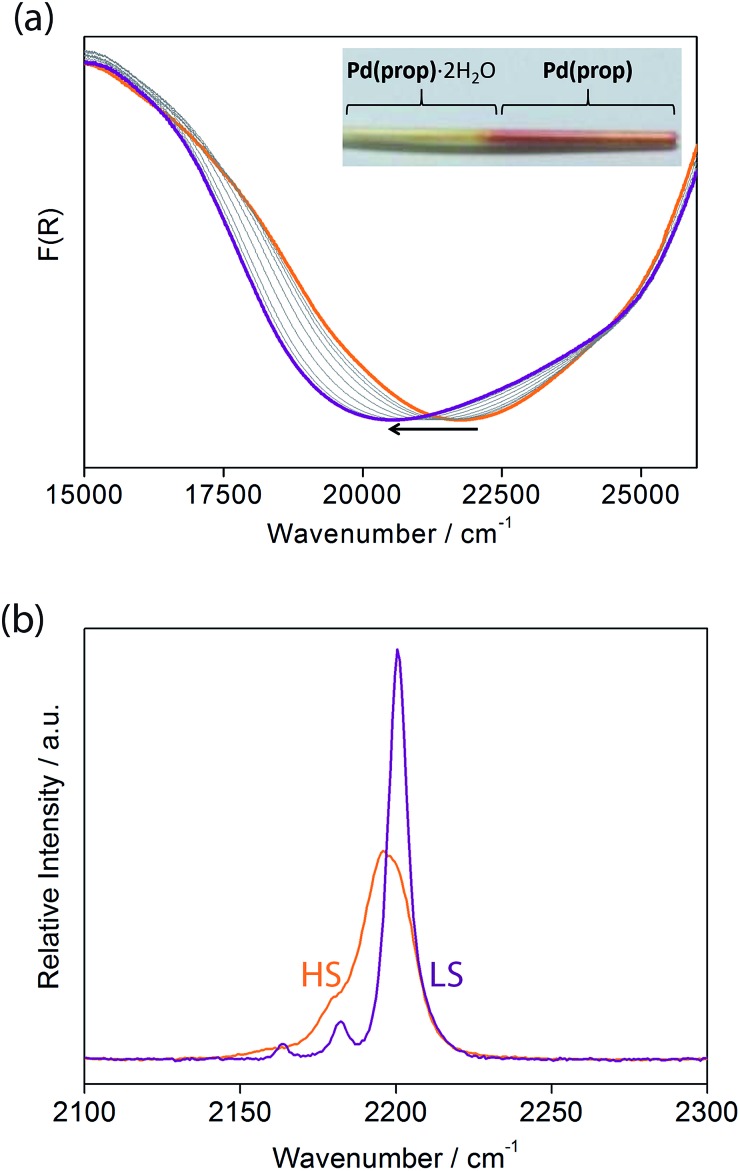
(a) Variable temperature solid state UV-vis spectra of **1(Pd)**·2H_2_O (orange) and **1(Pd)** (purple). The arrow indicates the direction of the spectral change with heating to the dehydrated material. Inset: photograph of a capillary showing the difference in colour of **1(Pd)**·2H_2_O (yellow) and **1(Pd)** (pink). (b) Observed Raman spectra of **1(Pd)** in the HS (orange) and LS (purple) states showing the difference in the alkyne and cyanide stretching bands.

Let us now consider some of these factors. Given that the ligands in the hydrated phases interact in pairs constrained by water molecule interactions and proximity, guest removal likely facilitates the optimisation of these ligand–ligand interactions into more aligned dimers or even towards 1D stacks (Fig. 2(b)). Such a transition to more effective host–host contacts would provide enhanced communication pathways for spin state propagation. Supporting this hypothesis, DFT calculations confirm that the ligands are more energetically free to rotate with removal of the host–guest hydrogen bonding interactions associated with the removal of guest water molecules (Fig. S28†). The relative importance of this structural change is further evident in an observed yellow to red shift in the optical absorption spectrum just above ambient temperature (*i.e.*, in the HS state) upon water removal (Fig. 5(a)). Time-dependent DFT calculations simulating these spectral changes confirm that a red shift occurs concomitant with more effective ligand–ligand interactions (Fig. S29†). Indeed, in unrelated systems such colour shifts have been known to correspond to a concentration of interactions such as aromatic interactions.^41^ Furthermore, in order to gain some insight into potential electronic effects of hydration, we have also used small model systems to directly simulate the SCO behaviours (Fig. S30†). Our results suggest that the binding of water to the ligand should indeed provide electronic stabilisation to the HS state relative to the LS state, thus further contributing to the observed difference in the SCO behaviour between the hydrated and dehydrated materials (Fig. S31†).

**Fig. 6:**
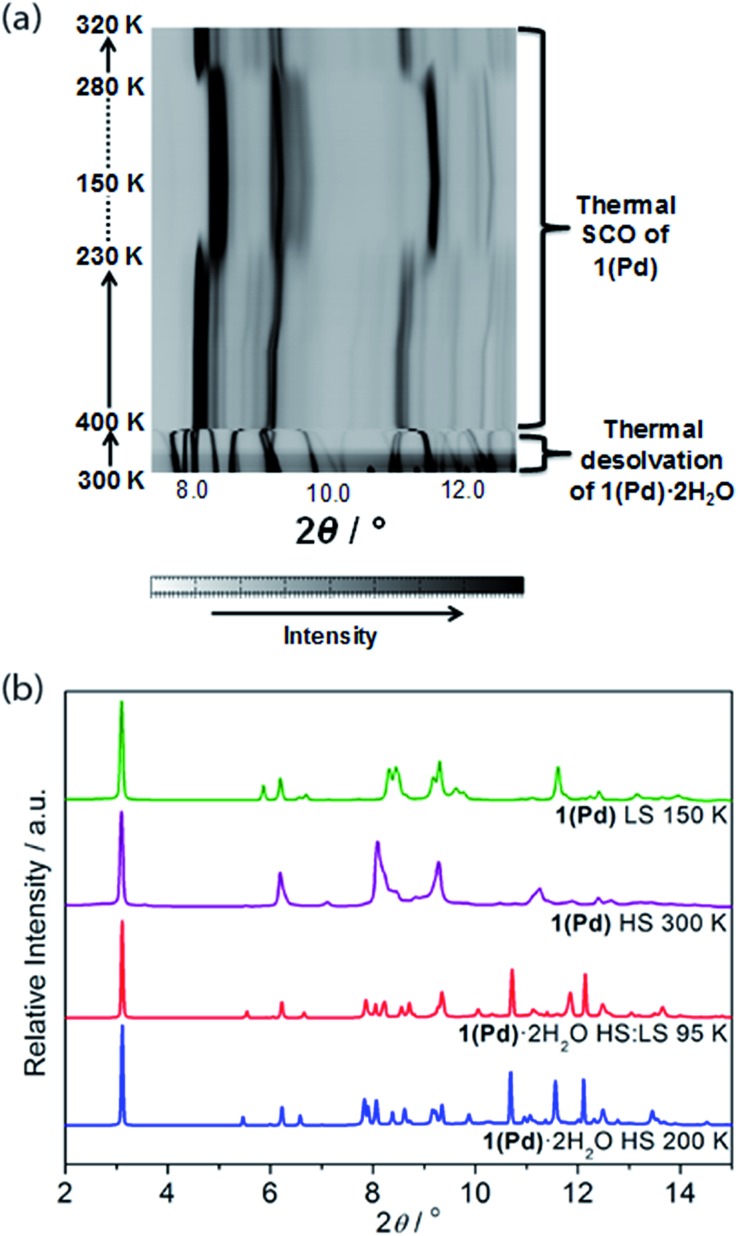
(a) Powder X-ray diffraction peak evolution for **1(Pd)** (8.0–12.5°) showing *in situ* dehydration and SCO as a function of temperature. (b) Comparison of powder X-ray diffraction data (2–15°) for **1(Pd)**·2H_2_O (at 200 and 95 K) and **1(Pd)** (at 300 and 150 K), highlighting structural changes, peak broadening, and peak overlap with guest removal and SCO.

*In situ* removal of guest water molecules from **1(Pd)**·2H_2_O during single crystal diffraction measurements led to a rapid loss of crystal integrity above ambient temperature. Thus, studies on the structural implications of guest removal were conducted *via in situ* powder X-ray diffraction using synchrotron radiation. With heating from room temperature to 330 K the structure remains unchanged (Fig. 6(a)). Above this temperature a rapid and irregular shift in peaks occurs. In comparing the powder diffraction patterns of **1(Pd)**·2H_2_O and **1(Pd)** at high temperature (200 K and 300 K, respectively), the dehydrated phase has broader and less well-resolved peaks but retains the characteristic intense low angle interlayer peak of Hofmann-type materials (Fig. 6(b)). Due to diffraction peak overlap and low (triclinic) symmetry, extracting unit cell information from this dehydrated HS phase was not possible. However, the diffraction peaks of the LS phase of **1(Pd)** are less broad and better resolved, enabling further structural analysis (Fig. 6(b)). (It must be noted that the crystallinity of **1(Pt)** significantly diminishes over the spin transition as measured by variable temperature powder diffraction, thus no considerable structural information could be obtained.)

This distinction between the diffraction patterns of **1(Pd)** in the HS and LS states offers some insight into the SCO behaviour as it suggests that there are notable structural differences between the two spin states of this material (Fig. 6(b) and S20†). Notably, our DFT calculations suggest that in the HS state, the Fe–N bond lengths are longer by *ca.* 0.25 Å than in the LS state, which is consistent with the general knowledge of the difference in Fe(ii) radii between the two spin states.^3^ In general, SCO accompanied by crystallographic phase changes or other significant structural variations create an energetic barrier for a spin transition, thus generating wide hysteresis loops.^42^–^44^ The dissimilarity between the HS and LS diffraction patterns also suggests that in adopting the LS state more structural order is achieved. Once structurally optimised in the LS state, the dehydrated phases are clearly resistant to reversion to the HS state, as reflected in the wide thermal hysteresis profiles.

Using Rietveld refinement methods, unit cell parameters were successfully extracted from the LS diffraction pattern of **1(Pd)**, revealing a cell similar to the hydrated phase but with notable modifications to the triclinic angles (Fig. S18, Table S4†). The unit cell changes indicate a range of subtle structural rearrangements, such as minor layer translations, consistent with modification of ligand–ligand interactions accompanying dehydration (Fig. 2(b) and S19†). Rietveld refinement carried out on the LS **1(Pd)** phase confirms that the overall Hofmann-type topology is retained with dehydration. The necessarily highly constrained nature of these refinements prevents the elucidation of finer structural details such as ligand geometry, including the alignment of face-to-face interactions of ligands and dimerisation of uniform stacks, and the degree of planarity of the Hofmann layer. However, the refinement model reveals average Fe–N bond lengths consistent with LS Fe(ii) sites (*d*_Fe1–N_ = 1.95 Å; Fig. S19, Table S5†).

We note that the retention of a triclinic unit cell in the dehydrated phases necessitates by symmetry that the ligand–ligand interactions retain a component of dimerisation, but, as discussed above, it is noteworthy that this appears to be reduced with respect to that of the parent hydrated phase, with removal of the hydrogen bonded water molecules leading to a more uniform 1D stack of ligand–ligand interactions along the *a*-axis (Fig. 2(b)). Efforts to further quantify the extent of this change through analysis of the vibrational spectra of the HS hydrated and dehydrated phases proved inconclusive due to the insensitivity of the primary ligand vibrational modes to crystal packing effects.

Considering that structural information of **1(Pd)** in the HS state could not be obtained due to partial degradation of crystallinity with dehydration, variable temperature Raman spectroscopy was performed to provide some insight into the vibrational details of the HS and LS states of **1(Pd)** (Fig. 5(b), S33†). To accompany this, DFT calculations were performed to obtain simulated spectra and to determine the vibrational modes of **1(Pd)** in both spin states (Tables S6 and S7†). Both spectra indicate a symmetrical alkyne stretching mode, which is slightly shifted to a higher frequency in the LS state (*ν*_C≡C_ = 2200 cm^–1^) compared to the HS state (*ν*_C≡C_ = 2196 cm^–1^). The cyanide stretch of the Hofmann layer in the LS state occurs as a well-resolved doublet corresponding to the symmetric and asymmetric stretching modes (*ν*_C≡N_ = 2163, 2182 cm^–1^), whereas there is only one weak band present as a shoulder in the HS spectrum corresponding to a symmetric cyanide stretch (*ν*_C≡N_ = 2180 cm^–1^) (Fig. 5(b)). Since both the symmetric and asymmetric cyanide stretches are Raman active in the LS state but only the symmetric cyanide stretch is Raman active in the HS state, this indicates a possible structural change with SCO in **1(Pd)**, complementing the powder diffraction data.

## Conclusions

In conclusion, this family of materials represents the first example of ambient temperature SCO with wide thermal hysteresis in 2D Hofmann-type frameworks. The array of variable temperature structural and magnetic measurements conducted on the guest-loaded (**1(Pd)**·2H_2_O and **1(Pt)**·2H_2_O) and the guest-free (**1(Pd)** and **1(Pt)**) frameworks indicate that magneto-structural lattice cooperativity is enhanced when strong intermolecular interactions are not only present but optimally distributed to proliferate a 3D communication of spin state information. In many other reported SCO materials, guest removal negatively impacts SCO cooperativity through the removal of host–guest interactions, thus disturbing the elastic interaction communication pathways. Here, despite the loss of these host–guest interactions with dehydration, the flexible nature of the interdigitated 2D Hofmann layers in the empty framework facilitates a concerted optimisation of ligand–ligand interactions. We also show that with the guest water molecules present, antagonistic interactions in the form of competing host–host and host–guest contacts act to elastically frustrate the lattice, causing stabilisation of a mixed spin state. Removal of these host–guest interactions in the guest-free phase eliminates the elastic frustration, resulting in a complete, one-step transition between the HS and LS states. Overall, this study shows that there is great potential for exploiting the intrinsic flexibility of 2D lattice topologies with strategically embedded interaction sites to generate further new examples of materials with bistable and ambient temperature electronic switching behaviours.

## Conflicts of interest

There are no conflicts of interest to declare.

## Supplementary Material

Supplementary information

Crystal structure data
