# Supplementary material for: Increasing spin crossover cooperativity in 2D Hofmann-type materials with guest molecule removal
Source: Chem Sci. 2018 May 29;9(25):5623–9. doi: 10.1039/c8sc01040d (PMC6048833; doi:10.1039/c8sc01040d)
Supplement: Supplementary file 1 [file SC-009-C8SC01040D-s001.pdf]

Electronic Supplementary Material (ESI) for Chemical Science.
This journal is © The Royal Society of Chemistry 2018

Supporting Information

Increasing spin crossover cooperativity in 2D Hofmann-type materials with guest molecule removal

Katrina A. Zenere, Samuel G. Duyker, Elzbieta Trzop, Eric Collet, Bun Chan, Patrick W. Doheny, Cameron J. Kepert* and Suzanne M. Neville*

CONTENTS

S1 – Synthesis
S2 – Single Crystal X-ray Diffraction
S3 – Powder X-ray Diffraction
S4 – Magnetic Susceptibility Measurements
S5 – Variable Temperature Solid State UV-Visible Spectroscopy
S6 – Density Functional Theory Calculations
S7 – Variable Temperature Raman Spectroscopy and DFT Calculations
S8 – Thermogravimetric Analysis
S9 – References

S1 – Synthesis

General

All reagents and solvents were purchased from Aldrich, Merck or A.K. Scientific and were used without further purification. (Iron(II) perchlorate hexahydrate was handled carefully and in small amounts to avoid any potential explosions.)

Synthesis of (*E*)-3-phenyl-*N*-(4*H*-1,2,4-triazol-4-yl)prop-2-yn-1-imine

4-Amino-1,2,4-triazole (98%, 3.0 g, 36 mmol) was added to phenylpropargylaldehyde (97%, 3.5 g, 27 mmol) and dissolved in ethanol (50 mL). Sulfuric acid (98%, 0.1 mL) was added and the solution was stirred and heated under reflux for 5 h. The solution was left to cool to room temperature and water (50 mL) was added with stirring. The solution was left overnight and further cooled on ice to precipitate. The precipitate was filtered, washed with water (10 mL) then cold ethanol (15 mL), and dried under vacuum. Recrystallisation from hot ethanol yielded a light brown precipitate of (*E*)-3-phenyl-*N*-(4*H*-1,2,4-triazol-4-yl)prop-2-yn-1-imine (proptrz) (yield 4.4 g, 22 mmol, 83%). **^{1}H NMR** (500 MHz, d_6-DMSO, ppm): δ = 9.11 (s, 2H), 8.61 (s, 1H), 7.66 (m, $^4J_{H-H}$ = 2.0 Hz, $^3J_{H-H}$ = 6.5 Hz, 2H), 7.57 (m, $^4J_{H-H}$ = 2.0 Hz, $^3J_{H-H}$ = 7.5 Hz, 1H), 7.50 (m, $^3J_{H-H}$ = 6.5 Hz, $^3J_{H-H}$ = 7.5 Hz, 2H). **^{13}C[^{1}H] NMR** (125 MHz, d_6-DMSO, ppm): δ = 142.5, 138.8, 132.2, 130.9, 129.1, 119.8, 98.2, 83.4. **IR** (cm^{-1}): 3132 (w), 3105 (w), 3005 (w), 2212 (s), 1577 (m), 1506 (s), 1303 (m), 1168 (s), 1054 (m), 926 (m), 771 (m), 761 (m), 691 (m), 621 (s). **UV-Vis-NIR** (cm^{-1}): λ_{max} = 17200 (sh), 23910 (sh), 31000, 33810, 39650, 43290 (sh). **ESI-MS** (ESI$^+$, m/z): calculated for $[M+H]^+$ $C_{11}H_9N_4$ 197.22, found 196.93. **Elemental analysis** calculated for [$C_{11}H_8N_4$ + EtOH] $C_{13}H_{14}N_4O$ (%): C 64.45, H 5.82, N 23.13; found (%): C 64.86, H 4.49, N 22.49. **MP** = 144.6–146.8 ˚C.

Synthesis of [$Fe(proptrz)_2Pd(CN)_4$]·2H_2O

Crystals were synthesised by slow diffusion using the vial-in-vial method, with proptrz (8 mg, 0.039 mmol) and potassium tetracyanidopalladate (6 mg, 0.020 mmol) in a small vial and iron(II) perchlorate hexahydrate (5 mg, 0.020 mmol) in a large vial. The small vial was placed inside the large vial and a solvent of 1:1 EtOH:H_2O (20 mL) was added slowly to avoid disturbing the reactants. The vials were left for *ca.* 3 weeks to grow yellow crystals of **1(Pd)**·2H_2O (yield 10 mg, 0.014 mmol, 73%). **IR** (cm^{-1}): 3648 (m), 3320 (br), 3223 (br), 3127 (br), 2202 (s), 2172 (s), 1578 (m), 1523 (s), 1318 (w), 1172 (m), 1065 (m), 758 (m), 688 (w), 623 (m), 417 (s). **UV-Vis-NIR** (cm^{-1}): λ_{max} = 23600 (sh), 26450 (sh), 30615, 33470, 38615, 42810 (sh). **Elemental analysis** calculated for [**1(Pd)**·2H_2O + EtOH] $C_{28}H_{26}FeN_{12}O_3Pd$ (%): C 45.39, H 3.54, N 22.69; found (%): C 45.01, H 2.98, N 22.93. **NOTE:** It is often

difficult to obtain accurate CHN elemental analysis data of porous framework materials due to uncertainty in their guest content at the time of measurement. Here, the additional EtOH guest molecule included in the calculation reflects the synthesis conditions using a solvent mixture of 1:1 EtOH:H_2O (the sample was slightly damp at the time of measurement). The stoichiometry **1(Pd)**·2H_2O was confirmed by single crystal and thermogravimetric analyses.

Synthesis of [Fe(proptrz)$_2$Pt(CN)$_4$]·2H_2O

Crystals were synthesised by slow diffusion using the vial-in-vial method, with proptrz (8 mg, 0.039 mmol) and potassium tetracyanidoplatinate (7 mg, 0.020 mmol) in a small vial and iron(II) perchlorate hexahydrate (5 mg, 0.020 mmol) in a large vial. The small vial was placed inside the large vial and a solvent of 1:1 EtOH:H_2O (20 mL) was added slowly to avoid disturbing the reactants. The vials were left for *ca.* 3 weeks to grow yellow crystals of **1(Pt)**·2H_2O (yield 12 mg, 0.015 mmol, 78%). **IR** (cm^{-1}): 3654 (w), 3645 (w), 3327 (br), 3212 (br), 3123 (br), 2202 (s), 2170 (s), 1636 (w), 1578 (m), 1523 (s), 1318 (m), 1172 (s), 1064 (s), 758 (w), 623 (w). **UV-Vis-NIR** (cm^{-1}): λ_{max} = 23085 (sh), 26580 (sh), 30400, 33550, 39650, 42850 (sh). **Elemental analysis** calculated for [**1(Pt)**·2H_2O + 2H_2O] $C_{26}H_{24}FeN_{12}O_4Pt$ (%): C 38.11, H 2.95, N 20.51; found (%): C 38.22, H 2.35, N 20.21. **NOTE:** It is often difficult to obtain accurate CHN elemental analysis data of porous framework materials due to uncertainty in their guest content at the time of measurement. Here, the additional H_2O guest molecules included in the calculation reflect the hydrophilic nature of this material. The stoichiometry **1(Pt)**·2H_2O was confirmed by single crystal and thermogravimetric analyses.

S2 – Single Crystal X-ray Diffraction

Single crystal X-ray diffraction (SCXRD) data of **1(Pd)**·2H_2O were collected at the Université de Rennes, France, at 200 K and 110 K (after fast cooling to 90 K and subsequent heating) on an Agilent Technologies SuperNova Single Source diffractometer with a micro-source, using a Cu-K_{α} (λ = 1.54184 Å) radiation source, and fitted with an EosS2 detector. For each of the two temperatures a new single crystal was measured. Crystals were mounted in solvent on a cactus needle attached to a goniometer head using a thin film of paratone oil, and were cooled to the required temperature prior to collection. Data collection, integration and reduction were performed using CrysAlisPro.[1] Structural solution was completed within SHELXS-97[2] and refined using SHELXL-97[3] within the X-Seed[4] user interface. All atoms (except hydrogen atoms and water oxygen atoms) were refined anisotropically and hydrogen atoms were fixed using the riding model. The highly disordered water oxygen atoms were refined over two positions and the hydrogen atoms on these oxygens were not included due to the uncertainty of their positions. Pore calculations were conducted using the CALC SOLV function within PLATON.[5] Structural refinement details and parameters are listed in Table S1, and bond length and angle details are provided in Table S2 and S3. Ortep diagrams of the asymmetric unit are provided in Fig. S1 and S2 with ellipsoids shown at 50% probability.

In **1(Pd)**·2H_2O at 200 K, the metal atoms (Fe1 and Pd1) are located on special positions within the unit cell, thus were refined with occupancy 0.5; all other atoms in the framework were refined with occupancy 1.0. Similarly, at 110 K, the Fe atoms (Fe1 and Fe2) are located on special positions within the unit cell, thus were refined with occupancy 0.5, all other atoms in the framework (including Pd1) were refined with occupancy 1.0. This is consistent with the thermogravimetric analysis where a loss of two H_2O molecules occurs prior to complete desolvation (Fig. S33).

As mentioned in the manuscript, single crystal structural analyses of **1(Pd)**·2H_2O show square planar Hofmann layers when viewed down the *c*-axis (Fig. S3(a)) and a subtle torsional twist of the triazole ring with respect to the rest of the ligand (Fig. S3(b)).

Precession images (*hk0* plane) obtained from SCXRD of **1(Pd)**·2H_2O collected at 200 K and 110 K are shown in Fig. S4. At 200 K (HS state), Bragg peaks along the *a** and *b** directions agree well with a parent cell of dimensions $a \approx 7.3$ and $b \approx 7.7$ Å; while at 110 K (HS:LS state), the emergence of weak satellite peaks along the *b** direction indicate symmetry breaking and doubling of the *b*-axis to $b \approx$ 15.0 Å. At 110 K, the HS and LS sites order in stripes down the *a*-axis as depicted in Fig. 4 and S5.

Table S1 Crystallographic data for **1(Pd)**·2H_2O collected in the HS and HS:LS states and **1(Pt)**·2H_2O collected in the HS state.

	**HS**	**HS:LS**	**HS**
**Framework**	[Fe(proptrz)$_2$Pd(CN)$_4$]·2H_2O		[Fe(proptrz)$_2$Pt(CN)$_4$] ·2H_2O
**Formula**	$C_{26}H_{20}FeN_{12}O_2Pd$		$C_{26}H_{20}FeN_{12}O_2Pt$
**MW / g mol^{-1}**	694.79		783.47
***T* / K**	200(2)	110(2)	200(2)
**Crystal System**	Triclinic	Triclinic	Triclinic
**Space Group**	*P*-1	*P*-1	*P*-1
**λ / Å (source)**	1.54184 (CuK_α)	1.54184 (CuK_α)	1.54184 (CuK_α)
Z	1	2	1
***a* / Å**	7.2613(2)	7.0836(3)	7.27720(10)
***b* / Å**	7.6635(3)	15.1242(6)	7.64320(10)
***c* / Å**	13.5746(5)	13.5688(6)	13.5928(3)
***α* / °**	95.988(3)	96.424(4)	95.997(2)
***β* / °**	98.931(3)	97.878(4)	98.598(2)
***γ* / °**	90.085(3)	90.090(4)	90.080(2)
***V* / Å^3**	742.04(5)	1430.70(11)	743.35(2)
**ρ_{calc} / g cm^{-3}**	1.555	1.613	1.750
**μ / mm^{-1}**	9.196	9.539	12.973
**Data/restraints/parameters**	2898, 0, 192	5555, 0, 380	3066, 0, 192
**R_1 [$I > 2\sigma(I)$, all data][a]**	0.0258, 0.0267	0.0331, 0.0388	0.0560, 0.0562
**wR_2 [$I > 2\sigma(I)$, all data][b]**	0.0684, 0.0690	0.0941, 0.0993	0.1544, 0.1545
**GoF**	1.014	1.057	1.083

[a] $R_1 = \Sigma||F_o| - |F_c||/\Sigma|F_o$. [b] w$R_2 = [\Sigma[w(F_o^2 - F_c^2)^2]/\Sigma[w(F_o^2)^2]]^{1/2}$.

Table S2 Selected bond lengths and angles of **1(Pd)**·$2H_2O$ and **1(Pt)**·$2H_2O$ at 200 K (HS state).

**Bond length or angle**	**1(Pd)**·$2H_2O$ **HS (200 K)**	**1(Pt)**·$2H_2O$ **HS (200 K)**
**<*d*(Fe1–N)> / Å[a]**	2.16	2.16
**Fe1–N3 (Fe–$N_{triazole}$) / Å**	2.198(2)	2.191(7)
**Fe1–N1 (Fe–$N_{cyanide}$) / Å**	2.141(2)	2.148(7)
**Fe1–N2 (Fe–$N_{cyanide}$) / Å**	2.1512(19)	2.144(7)
**O1WA(H)···N4 / Å**	2.801(6)	2.77(2)
**O1WB(H)···N4 / Å**	2.774(6)	2.79(2)
***Σ*(Fe1) / °[b]**	15.7	12.8
**N1–Fe1–N2 / °**	87.78(7), 92.22(7)	87.9(3), 92.1(3)
**N1–Fe1–N3 / °**	88.95(8), 91.05(8)	89.8(3), 90.2(3)
**N2–Fe1–N3 / °**	89.35(7), 90.65(7)	89.1(3), 90.9(3)
**Fe1–N1–C1 / °**	176.5(2)	177.3(8)
**Fe1–N2–C2 / °**	177.2(2)	176.0(8)
**Fe1–N3–N4 / °**	126.57(15)	126.4(5)
**Fe1–N3–C3 / °**	125.28(17)	125.9(6)

[a] Average Fe–N distance. [b] Octahedral distortion parameter calculated by sum of $|90 - \theta|$ for the twelve *cis* N–Fe–N angles in the octahedral geometry.

Table S3 Selected bond lengths and angles of **1(Pd)**·$2H_2O$ at 110 K (HS:LS state).

**Bond length or angle**	**1(Pd)**·$2H_2O$ **HS:LS (110 K)**	**Bond length or angle**	**1(Pd)**·$2H_2O$ **HS:LS (110 K)**
**<*d*(Fe1–N)> / Å**[a]	1.97	**<*d*(Fe2–N)> / Å**[a]	2.14
**Fe1–N5 (Fe–$N_{triazole}$) / Å**	2.000(2)	**Fe2–N9 (Fe–$N_{triazole}$) / Å**	2.172(3)
**Fe1–N1 (Fe–$N_{cyanide}$) / Å**	1.961(2)	**Fe2–N4 (Fe–$N_{cyanide}$) / Å**	2.137(3)
**Fe1–N2 (Fe–$N_{cyanide}$) / Å**	1.955(3)	**Fe2–N3 (Fe–$N_{cyanide}$) / Å**	2.124(3)
**O1WA(H)···N6 / Å**	2.747(5)	**O2WA(H)···N10 / Å**	2.815(5)
**O1WB(H)···N6 / Å**	2.796(6)	**O2WB(H)···N10 / Å**	2.717(7)
***Σ*(Fe1) / °**[b]	12.5	***Σ*(Fe2) / °**[b]	28.2
**N1–Fe1–N2 / °**	88.87(10), 91.13(10)	**N3–Fe2–N4 / °**	86.48(9), 93.53(9)
**N1–Fe1–N5 / °**	89.33(10), 90.67(10)	**N4–Fe2–N9 / °**	88.39(9), 91.61(9)
**N2–Fe1–N5 / °**	88.68(10), 91.32(10)	**N3–Fe2–N9 / °**	88.08(9), 91.92(9)
**Fe1–N1–C1 / °**	177.8(3)	**Fe2–N4–C4 / °**	176.0(3)
**Fe1–N2–C2 / °**	176.3(3)	**Fe2–N3–C3 / °**	173.5(3)
**Fe1–N5–N6 / °**	124.32(18)	**Fe2–N9–N10 / °**	126.44(19)
**Fe2–N5–C5 / °**	127.3(2)	**Fe2–N9–C16 / °**	125.7(2)

[a] Average Fe–N distance. [b] Octahedral distortion parameter calculated by sum of $|90 - \theta|$ for the twelve *cis* N–Fe–N angles in the octahedral geometry.

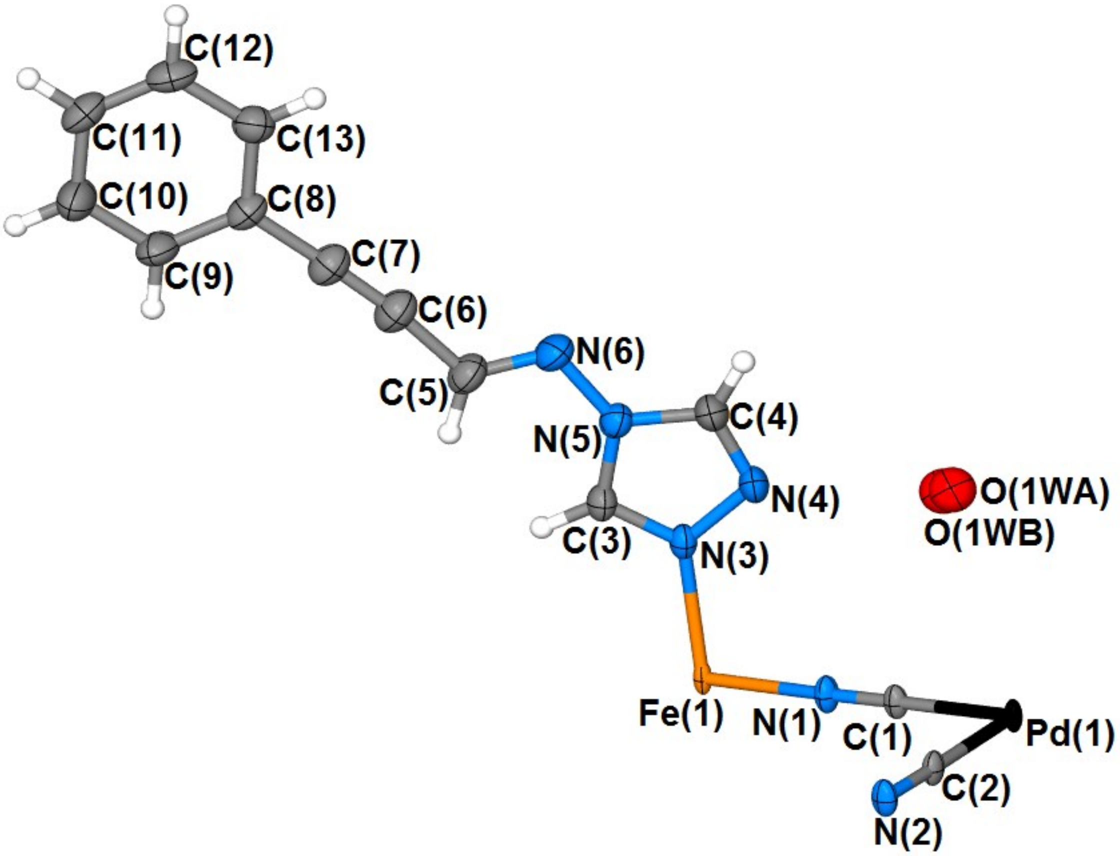

Fig. S1 Single crystal structure of **1(Pd)**·$2H_2O$ at 200 K (HS state) showing the asymmetric unit. Atoms are shown as thermal ellipsoids at 50% probability.

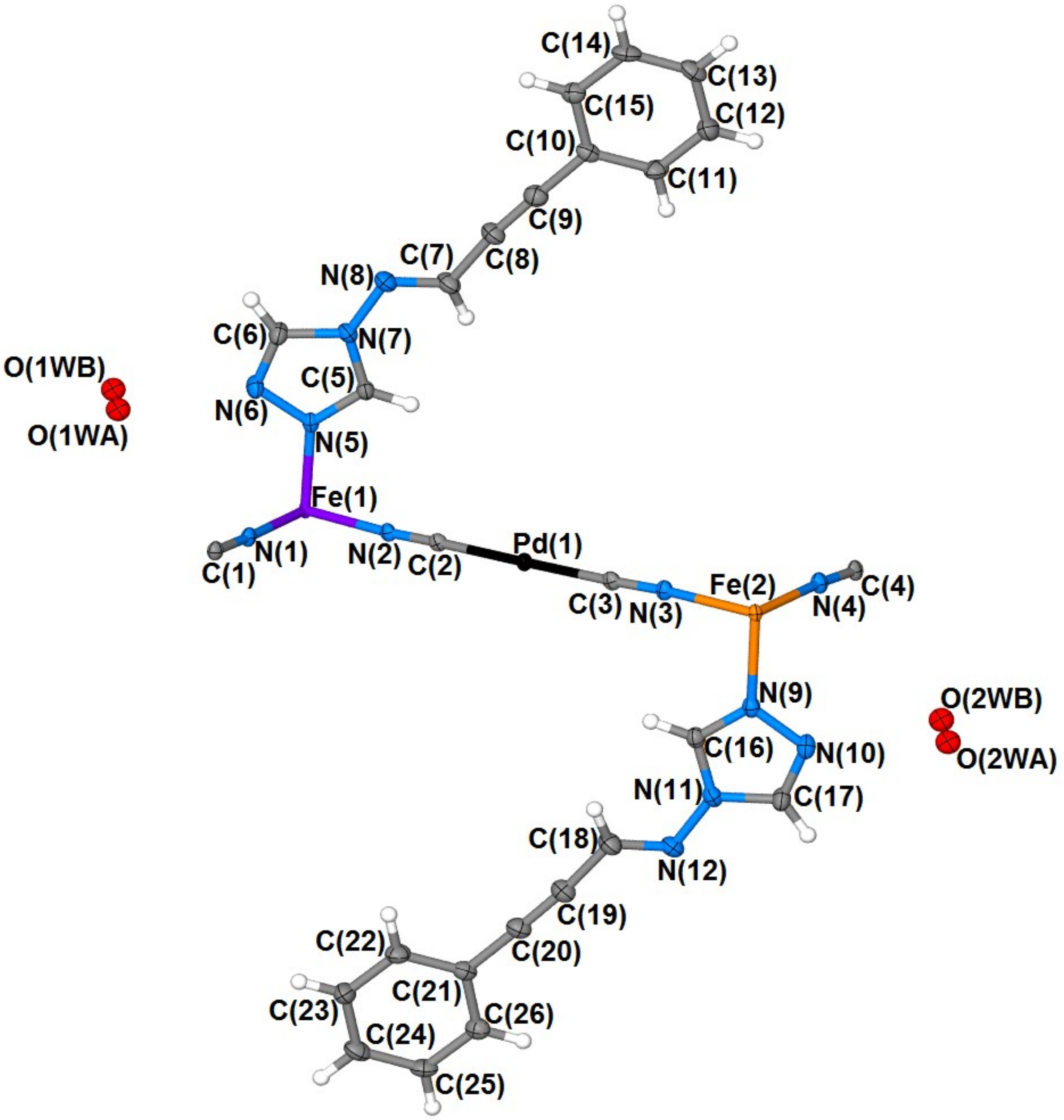

Fig. S2 Single crystal structure of **1(Pd)**·$2H_2O$ at 110 K (HS:LS state) showing the asymmetric unit. Atoms are shown as thermal ellipsoids at 50% probability.

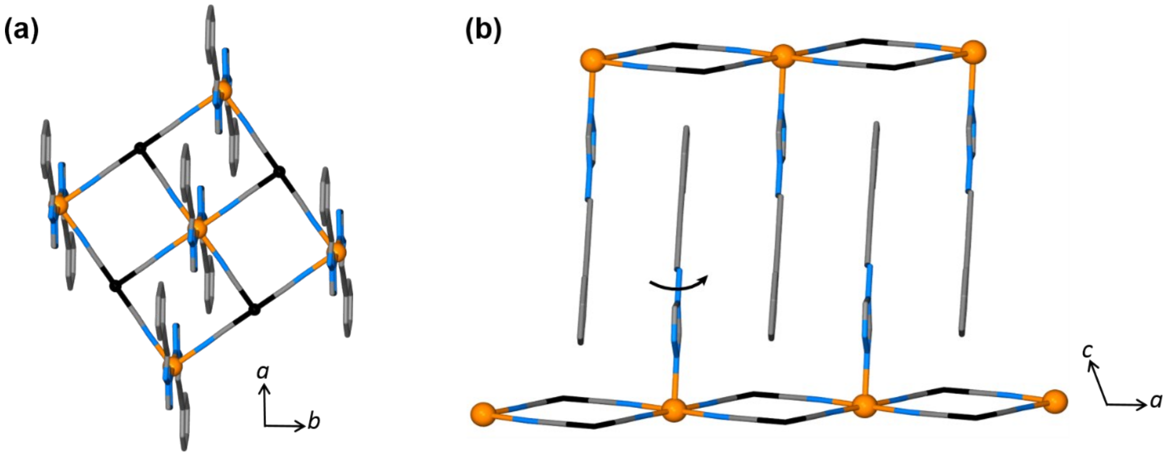

Fig. S3 Single crystal structure of **1(Pd)**·$2H_2O$ at 200 K highlighting the (a) square planar Hofmann layers and (b) subtle torsional twist of the triazole group compared to the rest of the proptrz ligand. Hydrogen atoms and water molecules have been omitted for clarity.

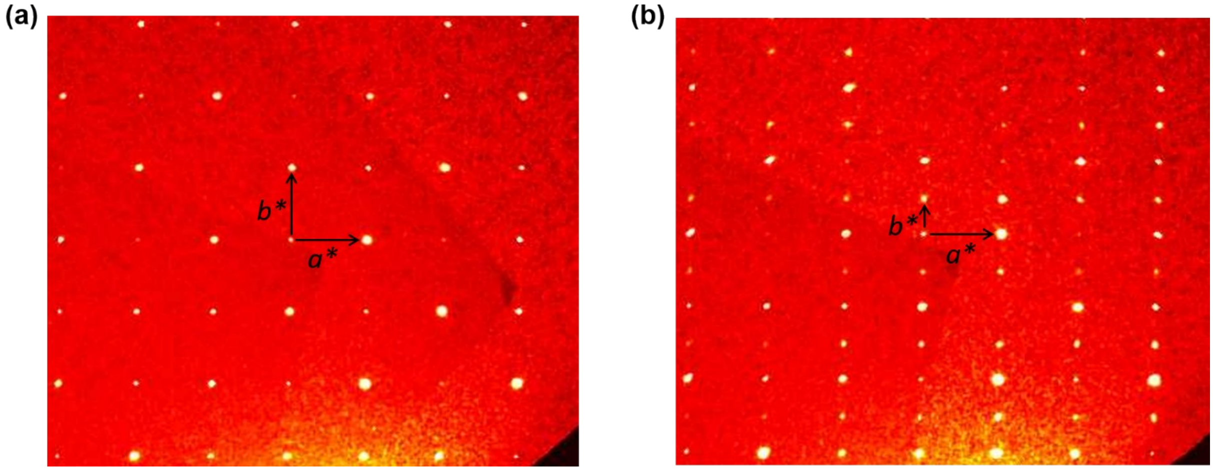

Fig. S4 Precession images of **1(Pd)**·$2H_2O$ for *hk0* at (a) 200 K (HS state) and (b) 110 K (HS:LS state). The emergence of satellite peaks corresponding to doubling of the *b*-axis are clearly evident at 110 K. (The halving of *b** indicates the doubling of the *b*-axis at 110 K compared to that at 200 K.)

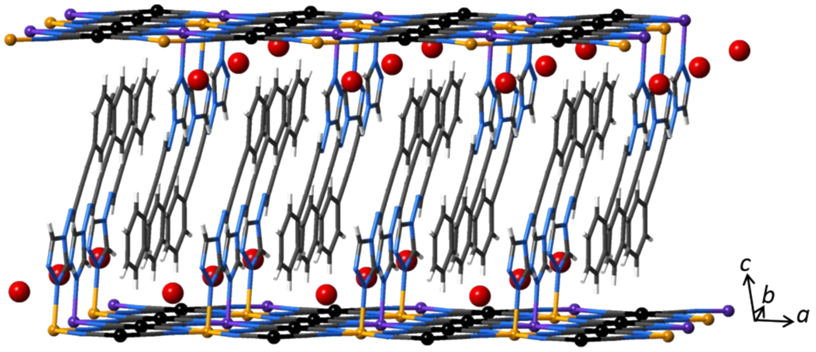

Fig. S5 Single crystal structure of **1(Pd)**·2H_2O at 110 K highlighting the ordering of the HS (orange) and LS (purple) sites in the *ac*-plane.

Single crystal diffraction data of **1(Pt)**·2H_2O were collected at the University of Sydney, Australia, at 200 K on an Agilent Technologies SuperNova Dual Source diffractometer using a Cu-K_α (λ = 1.54184 Å) radiation source, fitted with an Atlas CCD detector and an Oxford Cryostreams nitrogen cryostream. The crystal was mounted in solvent onto a loop attached to a goniometer head using a thin film of paratone oil, and was cooled to the required temperature (200 K) prior to collection. Crystal twinning made refinement of the cell-doubled low temperature (100 K) structure of the Pt analogue more difficult than the Pd analogue, so the HS:LS structure of **1(Pt)**·2H_2O will not be discussed. Data collection, integration and reduction were performed using CrysAlisPro.[1] The crystals show non-merohedral twinning intrinsic of the thin plate-like crystal morphology and this was accounted for in CrysAlisPro;[1] only the major twin component was used during refinements. Structural solution was completed within SHELXS-97[2] and refined using SHELXL-97[3] within the X-Seed[4] user interface. All atoms (except hydrogen atoms and water oxygen atoms) were refined anisotropically and hydrogen atoms were fixed using the riding model. The highly disordered water oxygen atoms were refined over two positions and the hydrogen atoms on these oxygens were not included due to the uncertainty of their positions. Structural refinement details and parameters are listed in Table S1, and bond length and angle details are provided in Table S2. An Ortep diagram of the asymmetric unit is provided in Fig. S6 with ellipsoids shown at 50% probability, and single crystal structural images are shown in Fig. S7.

For **1(Pt)**·2H_2O at 200 K, the metal atoms (Fe1 and Pt1) are located on special positions within the unit cell, thus were refined with occupancy 0.5, whereas all other atoms were refined with occupancy 1.0. The structural model of **1(Pt)**·2H_2O at 200 K includes two H_2O molecules within the pores per Fe site. This is consistent with the thermogravimetric analysis where a loss of two H_2O molecules occurs prior to complete desolvation (Fig. S33).

CCDC 1519487 (**1(Pd)**·2H_2O, 200 K, HS state), CCDC 1519488 (**1(Pd)**·2H_2O, 110 K, HS:LS state) and CCDC 1519489 (**1(Pt)**·2H_2O, 200 K, HS state) contain the supplementary crystallographic data for this paper. These data are provided free of charge by The Cambridge Crystallographic Data Centre.

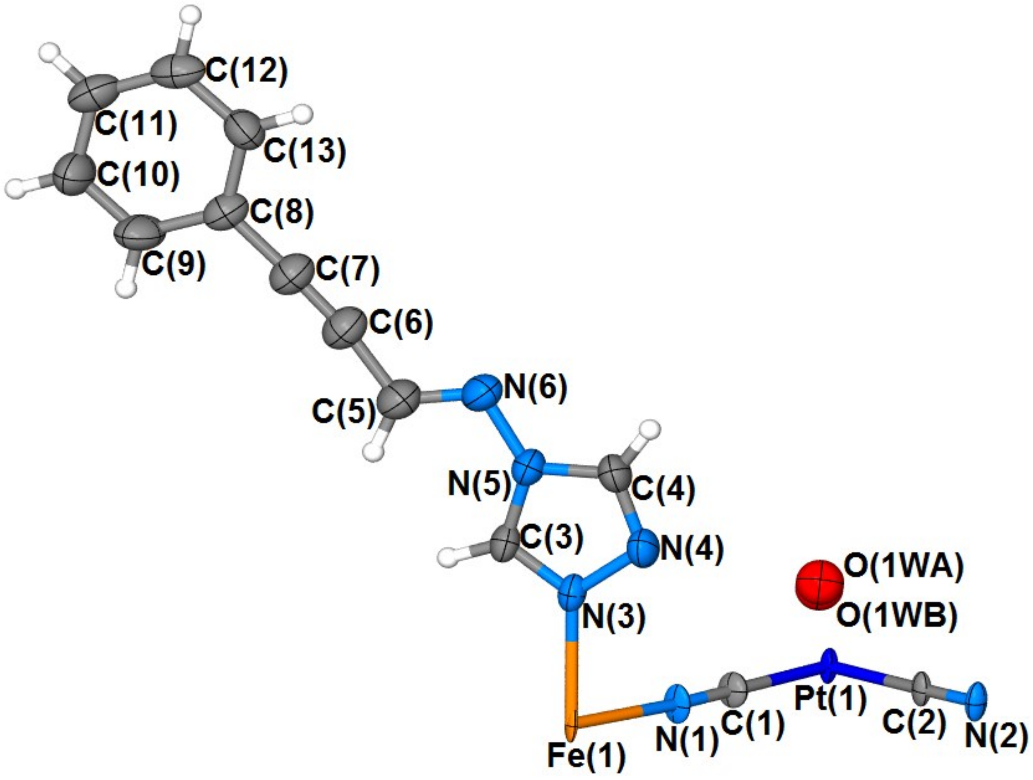

Fig. S6 Single crystal structure of **1(Pt)**·2H_2O at 200 K (HS state) showing the asymmetric unit. Atoms are shown as thermal ellipsoids at 50% probability.

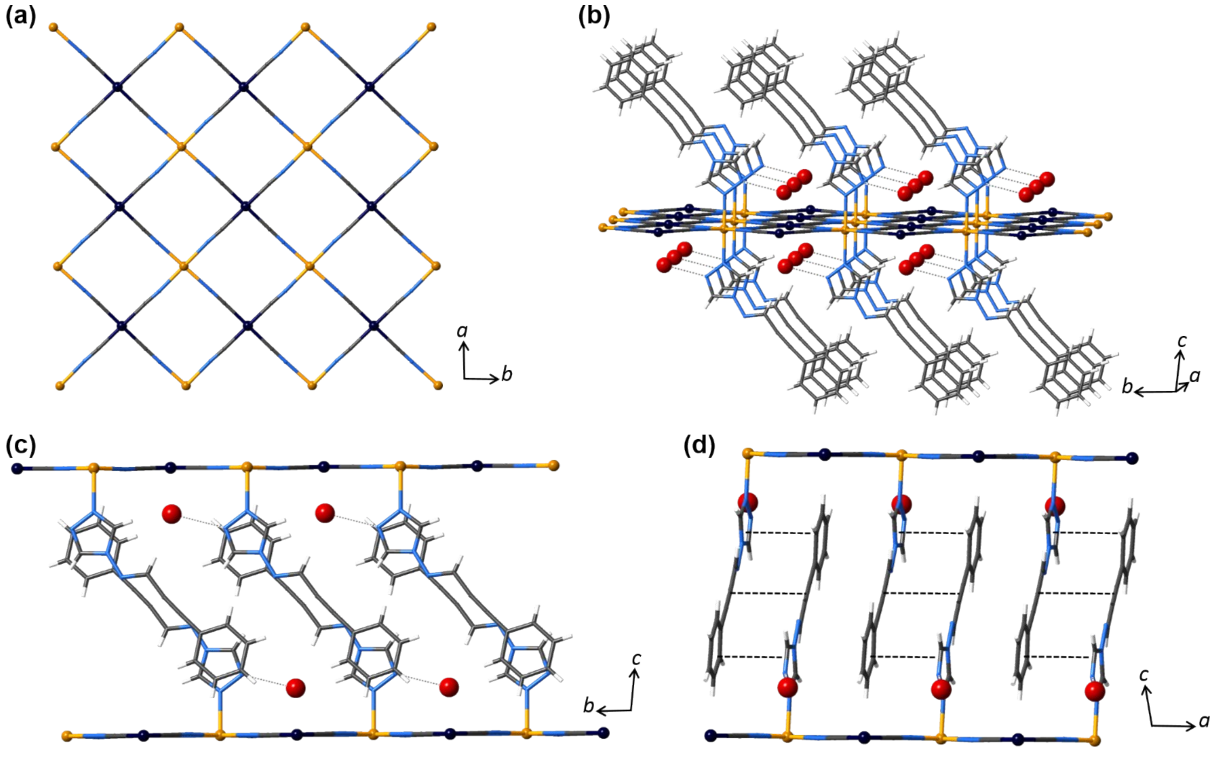

Fig. S7 Single crystal structure of **1(Pt)**·$2H_2O$ at 200 K showing (a) a square planar $[Pt(CN)_4]^{2-}$ Hofmann layer with a single crystallographically distinct Fe(II) site, (b) host–guest hydrogen bonding interactions, (c) interdigitation of the proptrz ligands from adjacent layers, and (d) aromatic interactions between pairs of ligands. Ligands and water guest molecules have been omitted in (a) for clarity, and the disordered water molecules have not been represented in (b–d) for clarity.

Additionally, variable temperature single crystal X-ray diffraction data of **1(Pd)**·$2H_2O$ were collected at the University of Sydney, Australia, using an Agilent Technologies SuperNova Dual Source diffractometer with a Mo-K_α (λ = 0.71073 Å) radiation source in the temperature range 200–100–200 K in 5 K intervals at a scan rate of 60 K h^{-1}. Data were refined in CrysAlisPro[1] using the parent cell (Fig. S8–S10). The data display the expected change in volume with SCO, namely a *ca.* 4% decrease in unit cell volume, as well as evidence of hysteresis, consistent with the magnetic susceptibility data. However, there is a slight deviation in the transition temperatures compared to the magnetic susceptibility measurements attributed to the different set-up conditions, such as methods of temperature control and measurement. The variable temperature evolution of the *a*- and *b*-axes and the β angle also match the magnetic susceptibility data since they decrease upon cooling and increase upon heating, each displaying thermal hysteresis. The *c*-axis and α angle show the opposite behaviour, increasing upon cooling and decreasing upon heating. This is not uncommon for triclinic systems, where the contraction of one unit cell parameter upon cooling may induce expansion of another unit cell parameter to compensate for the appropriate unit cell volume at that

temperature. Interestingly, the *γ* angle remains relatively constant throughout the entire SCO temperature range, indicating that the Hofmann layers retain their square planar geometry.

Variable temperature SCXRD data were unable to be collected on **1(Pt)**·$2H_2O$ as single crystallinity diminishes over the spin transition.

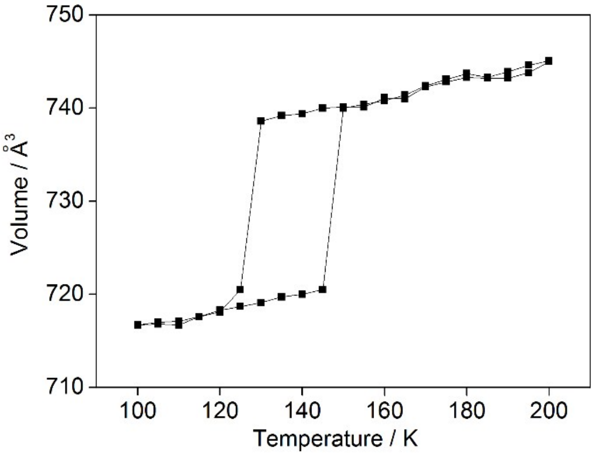

Fig. S8 Evolution of the unit cell volume of **1(Pd)**·$2H_2O$ using variable temperature SCXRD.

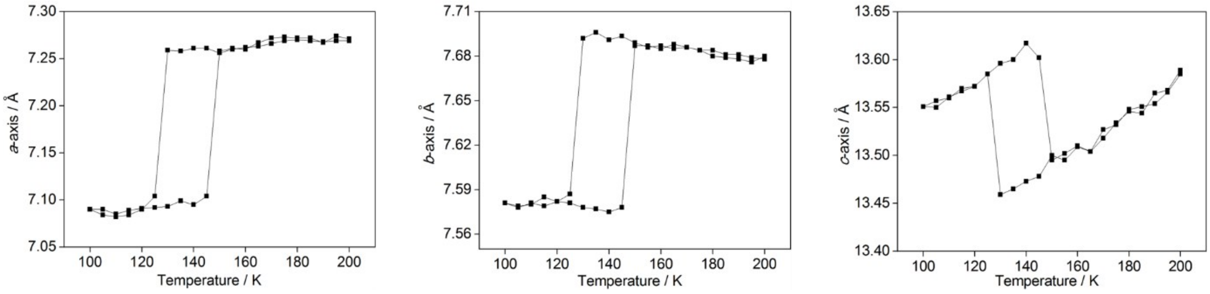

Fig. S9 Evolution of the *a*, *b* and *c* axes of **1(Pd)**·$2H_2O$ using variable temperature SCXRD.

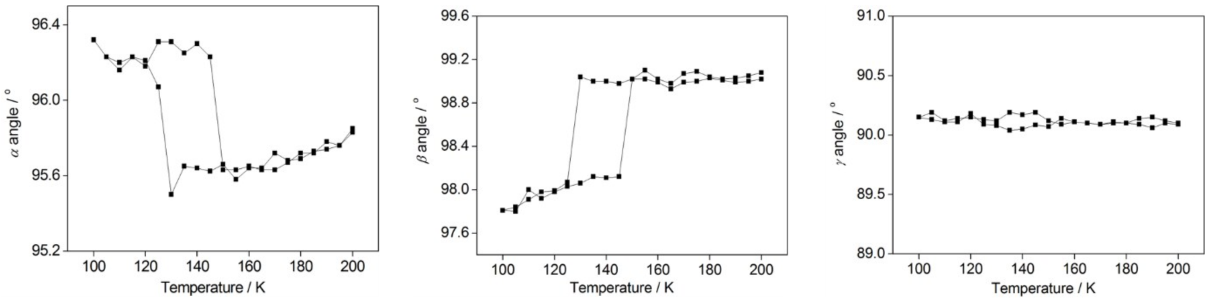

Fig. S10 Evolution of the *α*, β and *γ* angles of **1(Pd)**·$2H_2O$ using variable temperature SCXRD.

S3 – Powder X-ray Diffraction

Variable temperature synchrotron powder X-ray diffraction (PXRD) measurements were undertaken at the Advanced Photon Source (APS), Argonne National Laboratory, Chicago, USA, on the 17-BM beamline (λ = 0.72775 (hydrated), 0.72768 Å (dehydrated)). Measurements were conducted using a Perkin Elmer area detector at a distance of 800 mm (hydrated) or 500 mm (dehydrated) with 5–10 s exposures. $Na_2Ca_3Al_2F_{14}$ (NAC) was used as the standard. Experiments on both the hydrated and dehydrated materials were conducted:

1) Polycrystalline samples of **1(Pd)**·$2H_2O$ and **1(Pt)**·$2H_2O$ were ground as a slurry in a 1:1 EtOH:H_2O solvent, loaded into 0.7 mm glass capillaries and sealed. The sample temperature was controlled using an Oxford Cryostreams cryostat, and the data were collected over the temperature range 200–95–200 K with a continuous ramp rate of 120 K h^{-1}.
2) Polycrystalline samples of **1(Pd)**·$2H_2O$ and **1(Pt)**·$2H_2O$ were ground as a slurry in a 1:1 EtOH:H_2O solvent, and loaded into 0.7 mm glass capillaries that were left open at one end to enable *in situ* dehydration. The sample temperature was controlled using an Oxford Cryostreams cryostat. The data were collected with initial heating from 300 to 400 K (with a continuous ramp rate of 240 K min^{-1}) and held at 400 K for 1 h to promote dehydration. The sample was then quickly cooled to 300 K (at 240 K min^{-1}) and data were collected over the SCO temperature range (**1(Pd)**: 300–150–325 K; **1(Pt)**: 300–100–350 K) with a continuous ramp rate of 120 K h^{-1}. (**NOTE:** Since these materials readily loose crystallinity with desolvation, multiple *in situ* and *ex situ* methods of dehydration of **1(Pd)**·$2H_2O$ and **1(Pt)**·$2H_2O$ to **1(Pd)** and **1(Pt)**, respectively, were conducted. The data involving the *in situ* dehydration resulted in the higher quality diffraction patterns so have been reported here.)

Data were integrated using Fit-2D[6, 7] (hydrated) or GSAS-II[8] (dehydrated). Le Bail refinements were conducted using TOPAS[9] and Rietveld refinements were conducted using GSAS-II.[8]

With plotting peak position evolution *vs.* temperature of the PXRD data of **1(Pd)**·$2H_2O$ and **1(Pt)**·$2H_2O$ over the temperature range 200–95–200 K, there is a clear shift to higher 2ϑ values upon cooling, which indicates a HS to LS spin transition (Fig. S11, 2ϑ = 6.5–6.7°, *hkl* = [011]). For both materials, the abruptness of the peak shift and the spin transition temperatures ($T_{½}{\downarrow} \approx$ 100 K, $T_{½}{\uparrow} \approx$ 130 K) correlate well with the magnetic susceptibility measurements. Although **1(Pt)**·$2H_2O$ does indeed diffract, it has a decreased resolution compared that of to **1(Pd)**·$2H_2O$, such that the crystallinity diminishes over the spin transition; thus, structural details and refinements are limited to the Pd analogue.

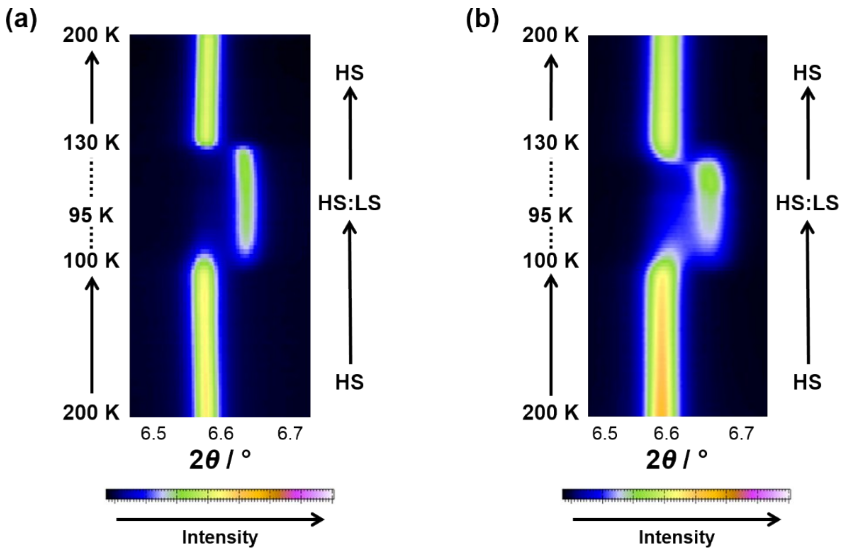

Fig. S11 Variable temperature PXRD peak evolution of (a) **1(Pd)**·$2H_2O$ and (b) **1(Pt)**·$2H_2O$ showing the shift of a single Bragg peak over the spin transition.

To accurately determine the thermal unit cell evolution of **1(Pd)**·$2H_2O$, Le Bail refinements (triclinic, *P*-1) were carried out on all diffraction patterns at the various temperatures collected and were modelled using the parent cell. Representative powder diffraction patterns for the HS (200 K) and HS:LS state (95 K) are provided (Fig. S12, Table S4). For all data, the calculated fits agree well with the experimental patterns, verifying bulk crystalline purity and the correct resultant unit cell. Plots of unit cell parameters as a function of temperature are provided and show similar behaviour to the SCXRD data (Fig. S13–S15). It must be noted that no weak satellite peaks indicting a unit cell with a doubled axis were observed at any temperature, thus all refinements were performed using the parent cell. Additionally, these experimental diffraction patterns of the HS and HS:LS states agree well with the simulated patterns determined from single crystal diffraction analysis (Fig. S16).

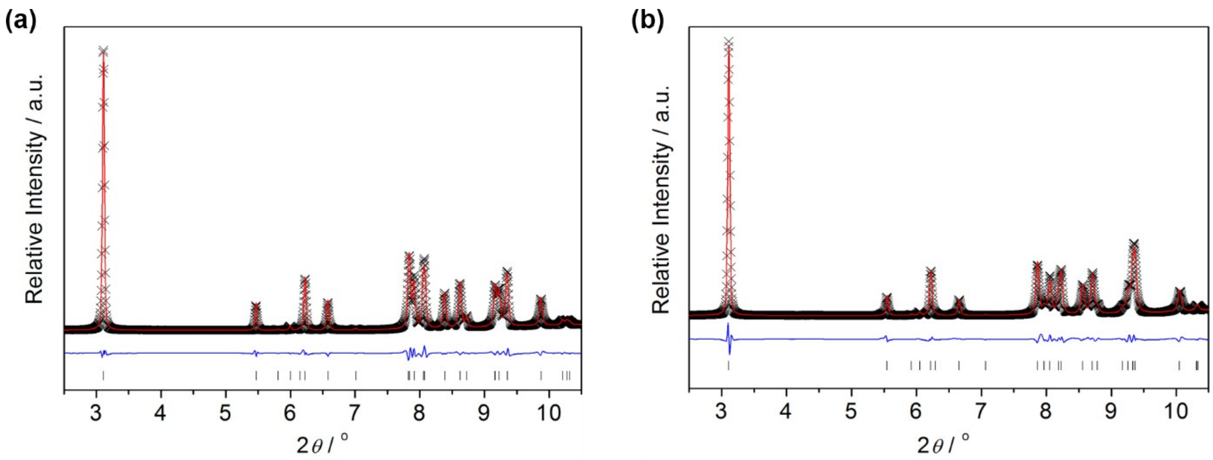

Fig. S12 Le Bail refinement of the PXRD patterns of **1(Pd)**·$2H_2O$ at (a) 200 K (HS state) and (b) 95 K (HS:LS state); experimental pattern (black crosses), calculated fit (red), difference (blue), *hkl* indices (black tick marks).

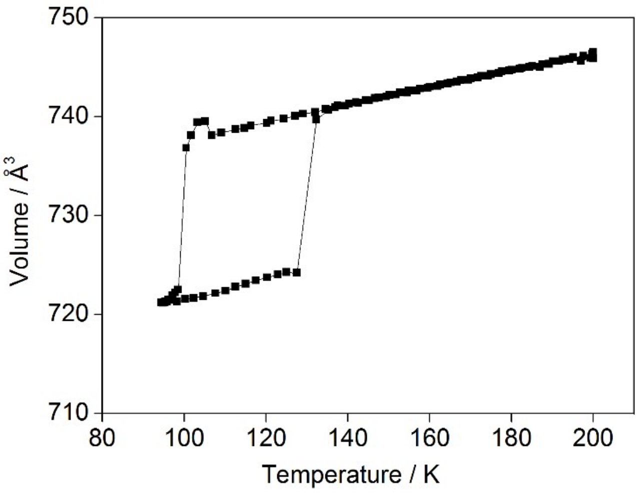

Fig. S13 Evolution of the unit cell volume of **1(Pd)**·$2H_2O$ using variable temperature PXRD.

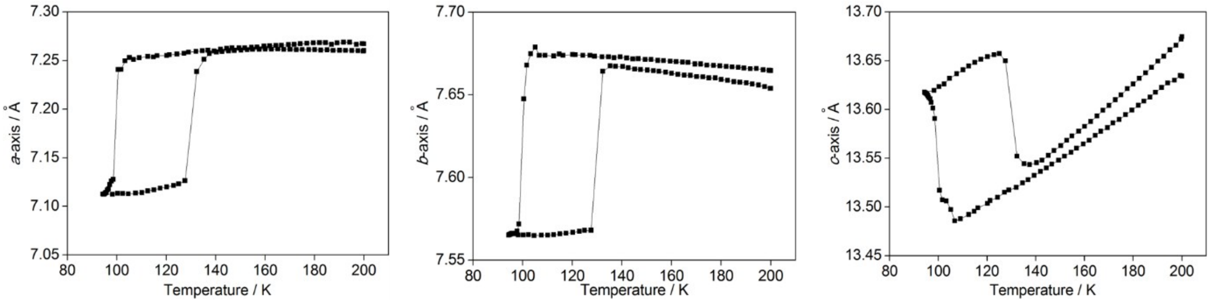

Fig. S14 Evolution of the *a*, *b* and *c* axes of **1(Pd)**·$2H_2O$ using variable temperature PXRD.

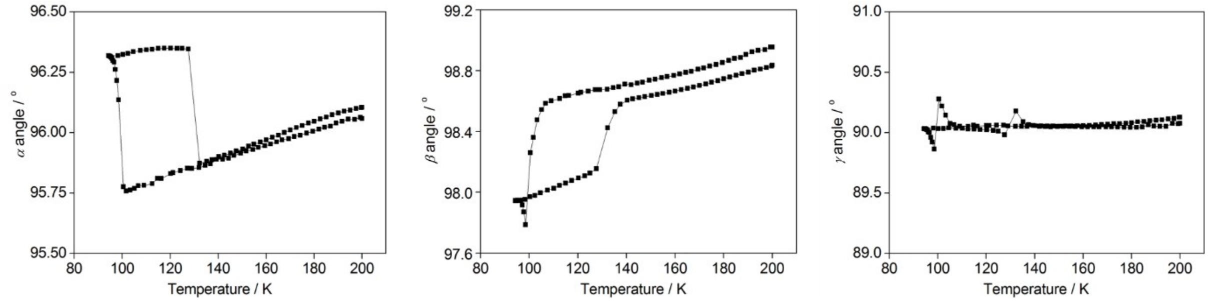

Fig. S15 Evolution of the *α*, β and *γ* angles of **1(Pd)**·$2H_2O$ using variable temperature PXRD.

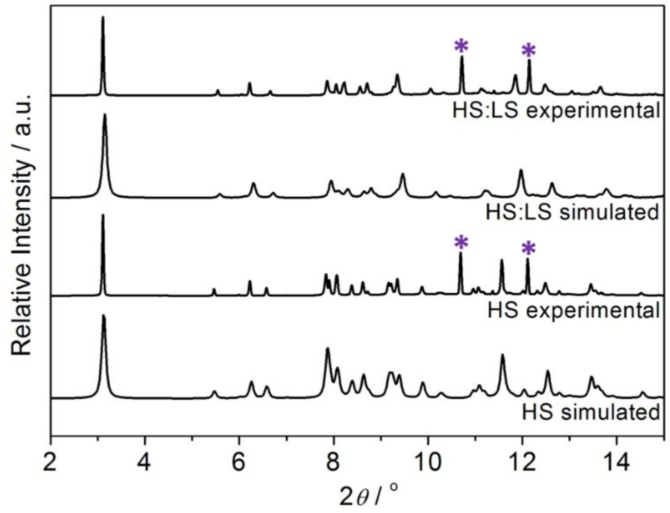

Fig. S16 Comparison of simulated and experimental diffraction patterns of **1(Pd)**·$2H_2O$ at 200 K (HS state) and 95 K (HS:LS state). Note that asterisks in the experimental patterns indicate impurities that were excluded during refinements.

Variable temperature PXRD measurements performed on **1(Pd)** display a shift in Bragg peaks with temperature variation (Fig. S17(a), 2ϑ = 8.0–8.6°). Measurements (300–150–325 K, 120 K h^{-1}) show a single-step, abrupt spin transition, with transition temperatures of *ca.* 240 and 270 K upon cooling and heating, respectively, which correlate well with the magnetic susceptibility measurements. Similarly, variable temperature PXRD measurements performed on **1(Pt)** (300–100–350 K, 120 K h^{-1}) show a shift in Bragg peaks correlating to an abrupt, single-step SCO with transition temperatures of *ca.* 240 and 270 K upon cooling and heating, respectively (Fig. S17(b), 2ϑ = 8.0–8.6°). These correlate well with the magnetic susceptibility measurements. However, **1(Pt)** does not diffract very well and its crystallinity diminishes over the spin transition, which makes indexing and Le Bail analysis difficult. Thus, no Rietveld refinement was conducted and no structural information could be obtained from these data.

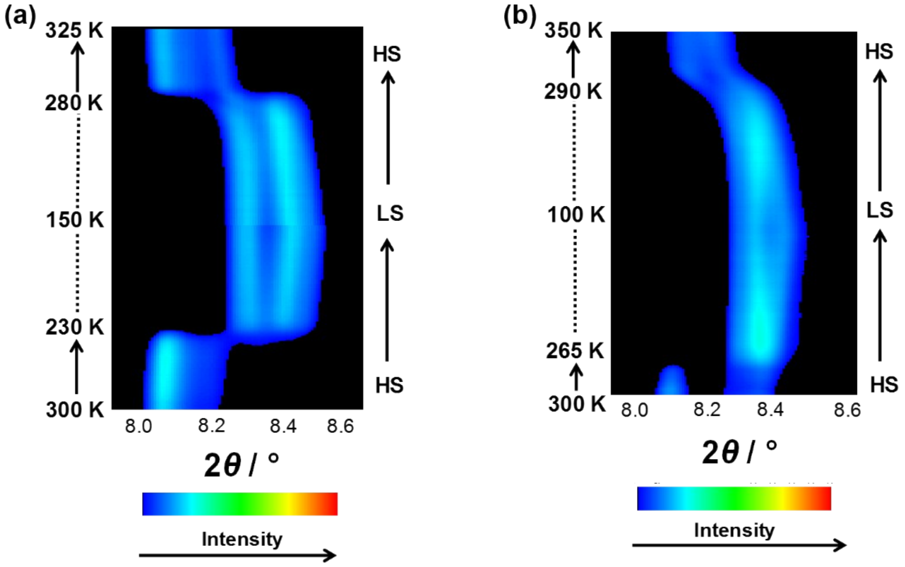

Fig. S17 Variable temperature PXRD peak evolution of (a) **1(Pd)** and (b) **1(Pt)** showing the shift of a single Bragg peak.

Rietveld refinement was conducted on the LS (150 K) diffraction pattern of **1(Pd)** (Fig. S18) using a rigid body model of the ligand obtained from single crystal analysis, with the atoms fixed but the position of this rigid body allowed to refine freely around the Fe–N bond. Atomic displacement parameters for the rigid body were treated using a single U_{iso} value. Analysis indicates the retention of the triclinic *P*-1 symmetry but with slightly different unit cell parameters compared to the hydrated phase (Table S4, Fig. S19). The Rietveld refinement indicates an average Fe–N bond length of 1.95 Å, consistent with LS Fe(II) at this low temperature (Fig. S19). The fractional coordinates and U_{iso} values for each atom in this model are provided in Table S5. A starting model for this constrained refinement was obtained through energy-minimisation DFT methods.

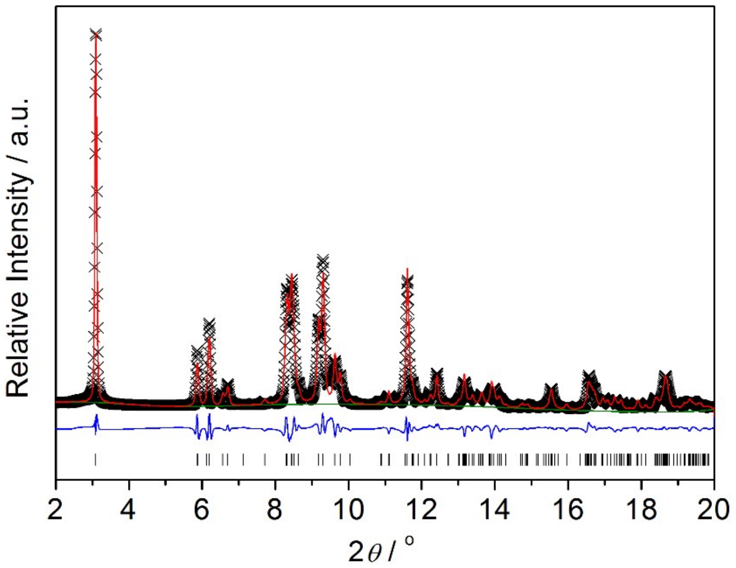

Fig. S18 Rietveld refinement of **1(Pd)** at 150 K (LS state); experimental pattern (black crosses), calculated fit (red), background (green), difference (blue), *hkl* indices (black tick marks). (Weighted residual: w*R* = 9.711%.)

Table S4 Crystallographic data for **1(Pd)**·$2H_2O$ and **1(Pd)** from powder diffraction.

	**1(Pd)**·$2H_2O$[a]	**1(Pd)**·$2H_2O$[a]	**1(Pd)**[b]
***T* / K**	200	95	150
**Spin state**	HS	HS:LS	LS
**Crystal system**	Triclinic	Triclinic	Triclinic
**Space group**	*P*-1	*P*-1	*P*-1
***a* / Å**	7.27	7.11	7.21
***b* / Å**	7.66	7.57	7.11
***c* / Å**	13.34	13.62	13.70
***α* / °**	96.06	96.32	91.35
***β* / °**	98.95	97.95	100.68
***γ* / °**	90.08	90.03	90.02
***V* / Å^3**	745.84	721.19	689.89

[a] Determined from Le Bail analysis. [b] Determined from Rietveld analysis.

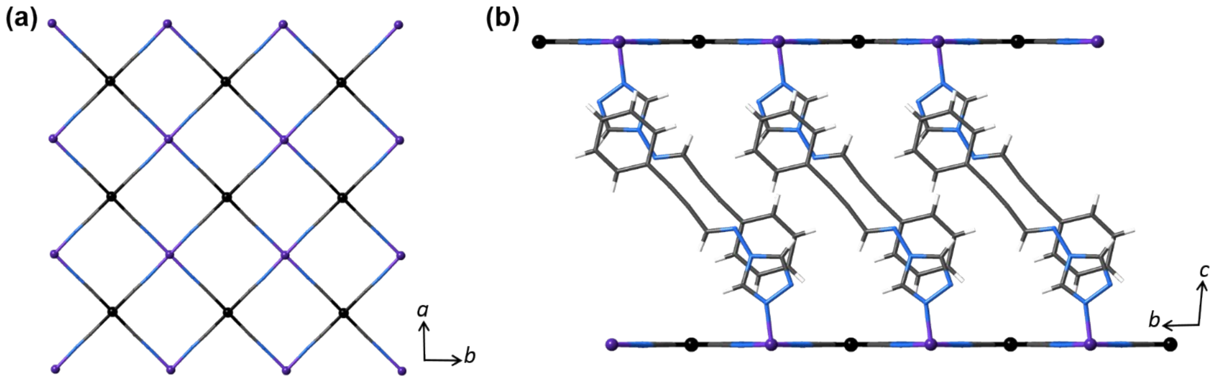

Fig. S19 Structural illustrations of **1(Pd)** in the LS state at 150 K, obtained from Rietveld refinement, showing the possible (a) square planar Hofmann grids and (b) interdigitation of ligands. Ligands were omitted from (a) for clarity.

Table S5 Structural details determined by the Rietveld analysis of powder diffraction data of LS **1(Pd)** at 150 K.

Atom	Multiplicity	Fractional Coordinates			Atomic Displacement Parameter (U_{iso})/10^{-2} A^2
		x	*y*	*z*	
Fe1	1	1	−1	1	3.36
Pd1	1	1.5	−0.5	1	7.28
N1	2	1.1994	−0.809	0.997	15.51
N2	2	1.1954	−0.1951	0.997	15.17
C1	2	1.3069	−0.6938	0.999	14.04
C2	2	1.304	−0.304	0.9988	12.74
N3	2	0.958157	−1.03805	0.862073	9.54
N4	2	0.937589	−0.88525	0.801992	9.54
C3	2	0.923197	−1.18832	0.804628	9.54
H3	2	0.92643	−1.3138	0.827008	9.54
C4	2	0.897868	−0.94884	0.710486	9.54
H4	2	0.882468	−0.87273	0.65351	9.54
N5	2	0.881634	−1.13812	0.708108	9.54
N6	2	0.845092	−1.24515	0.61696	9.54
C5	2	0.806725	−1.41998	0.626784	9.54
H5	2	0.805817	−1.46679	0.691112	9.54
C6	2	0.766008	−1.54281	0.542712	9.54
C7	2	0.726675	−1.65698	0.477477	9.54
C8	2	0.687116	−1.80239	0.404212	9.54
C9	2	0.652119	−1.75787	0.298616	9.54
H9	2	0.653996	−1.63195	0.277048	9.54
C10	2	0.615201	−1.91199	0.230039	9.54
H10	2	0.592468	−1.88624	0.161141	9.54
C11	2	0.610462	−2.09285	0.257031	9.54
H11	2	0.583554	−2.19147	0.208418	9.54
C12	2	0.646149	−2.13195	0.358645	9.54
H12	2	0.650377	−2.25889	0.379134	9.54
C13	2	0.674831	−1.99111	0.427832	9.54
H13	2	0.68713	−2.02211	0.495768	9.54

Although unit cell parameters and atomic positions of **1(Pd)** in the HS state could not be obtained, a speculation of the structure compared to the LS state can be made. Close examination of the powder diffraction patterns of **1(Pd)** reveal the brief existence of additional very weak diffraction peaks at low angles that are only present in the HS state (Fig. S20(a)). These modulation peaks indicate a doubling along either the *a*- or *b*-axis, such that both a structural and phase change takes place with dehydration and SCO.[10] It is suspected that this doubling is caused by one crystallographically unique Fe(II) site in **1(Pd)**·$2H_2O$ splitting into two distinct Fe(II) sites with desolvation to **1(Pd)** in the HS state (Fig. S20(b)). This then transforms back to a single unique Fe(II) site in the LS state without the doubled axis (i.e, the parent cell), after which the doubled cell is restored in the HS state when the spin transition is complete. A possible explanation is that this is a cooperative transition between the two Fe(II) sites in the HS state, where both sites undergo SCO at the same time as a result of strong ferro-elastic interactions. Such high cooperativity is demonstrated in the very wide hysteresis loop of **1(Pd)**. However, without indexing and refining these high temperature diffraction patterns no definite conclusion of the HS structure of **1(Pd)** can be made.

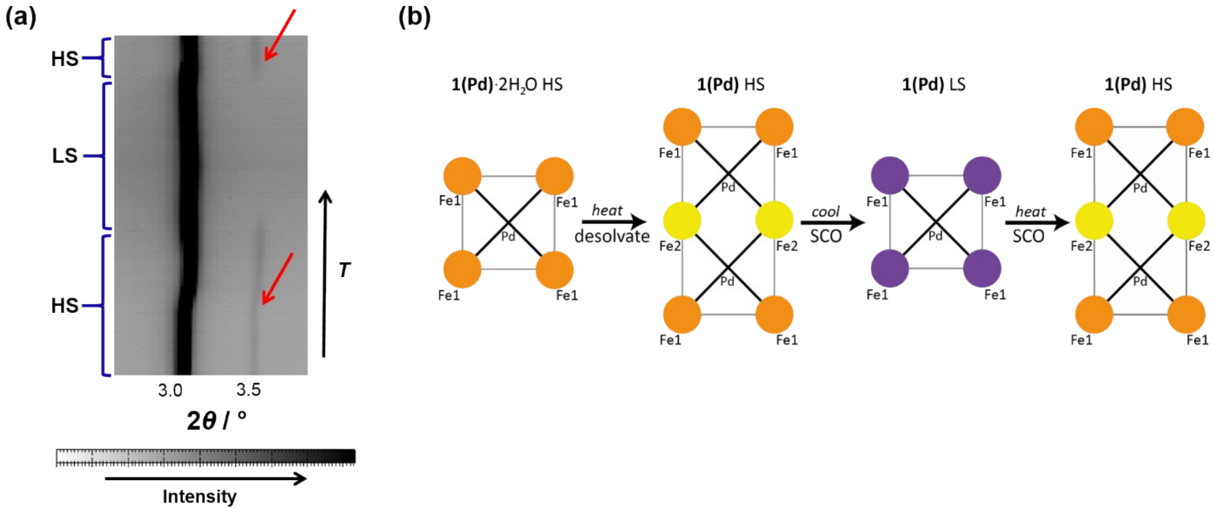

Fig. S20 (a) Variable temperature powder X-ray diffraction peak evolution (2ϑ = 2.8–4.0°) of **1(Pd)** showing evidence of modulation peaks in the HS state only. (b) Schematic of the desolvation and SCO processes of **1(Pd)** within the unit cell when viewed down the *c*-axis (i.e., the Hofmann layer), illustrating the possible HS (orange and yellow) and LS (purple) spin state arrangement and difference in crystallographically unique Fe(II) sites in the different spin states.

As a comparison of the loss in crystallinity with dehydration, diffraction patterns of **1(Pt)**·$2H_2O$ in the HS and HS:LS states, and **1(Pt)** in the HS and LS states are provided (Fig. S21). Not only do diffraction peaks lose intensity over the spin transition, but also become very broad (with peak overlap) with guest removal. Thus, structural information of **1(Pt)** at any temperature could not be obtained.

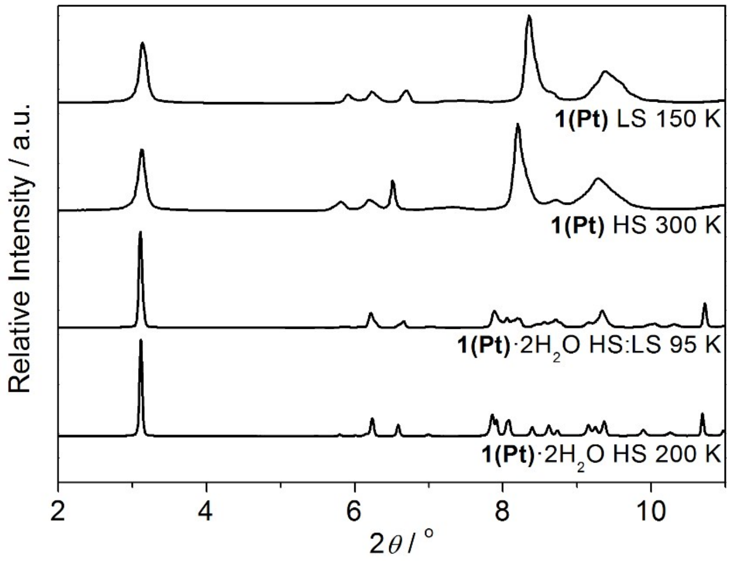

Fig. S21 Variable temperature powder diffraction patterns of **1(Pt)**·$2H_2O$ at 200 K (HS state) and 95 K (HS:LS state), and **1(Pt)** at 300 K (HS state) and 150 K (LS state), highlighting peak broadening and overlap with guest removal.

S4 – Magnetic Susceptibility Measurements

Magnetic susceptibility data were collected on a Quantum Design VersaLab magnetometer equipped with a Vibrating Sample Magnetometer (VSM) attachment, under an applied magnetic field of 0.3 T, over the appropriate SCO temperature range. Most of the measurements were taken continuously in sweep mode at a scan rate of 2 K min^{-1}. For comparison, settle mode measurements on samples of **1(Pd)**·2H_2O and **1(Pt)**·2H_2O were also performed in either 2 K increments (around the hysteresis) or 4 K increments (around the HS and LS plateau regions) with a 2 K min^{-1} scan rate in between each measurement (Fig. S22). Regardless of scan mode (sweep or settle), at equivalent rates we find no significant difference in the SCO behaviour, as was the case in our recent *J. Am. Chem. Soc.* publication on a similar 2D Hofmann-type framework material, [Fe(bztrz)$_2$Pd(CN)$_4$]·*n*(guest);[11] thus, the majority of the measurements reported here were performed in sweep mode as the experiments take considerably less time than when conducted in settle mode (i.e., 4 h as opposed to 24 h). (Note that magnetic susceptibility measurements of the dehydrated phases **1(Pd)** and **1(Pt)** could not be conducted in settle mode as these measurements are so slow the samples rehydrate in the magnetometer.) Data were also checked for reproducibility and variability by assessing: several successive cycles, a range of scan rates (1, 2 and 4 K min^{-1}), and a number of different batch samples of the same framework material.

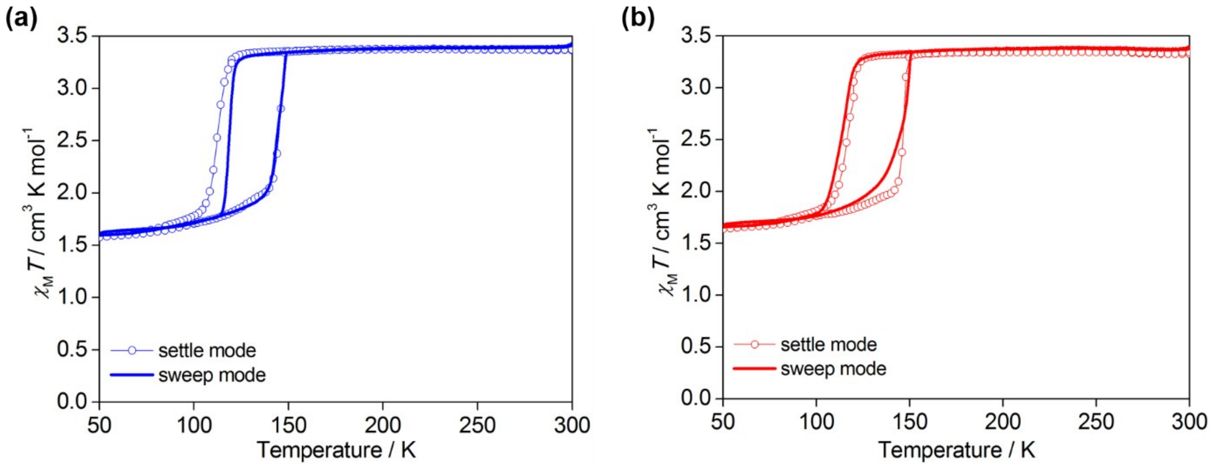

Fig. S22 Variable temperature magnetic susceptibility showing a comparison of (a) **1(Pd)**·2H_2O and (b) **1(Pt)**·2H_2O in sweep and settle modes measured at a scan rate of 2 K min^{-1}.

Magnetic susceptibility experiments were conducted on the following different phases:

1) Polycrystalline samples of **1(Pd)**·$2H_2O$ and **1(Pt)**·$2H_2O$ were washed with fresh 1:1 EtOH:H_2O solvent and loaded into a polypropylene (Formolene® 4100N) sample holder that snaps into a brass half-tube for measurement. To prevent solvent loss, Parafilm was wound around the join of the two halves of the polypropylene sample holder. The polypropylene sample holder and brass half-tube were the appropriate size for the small bore VSM option. Measurements were taken continuously in sweep mode at a scan rate of 2 K min^{-1} over the temperature range 300–50–300 K (Fig. 3(a)).
2) Dehydrated samples were prepared *in situ* by heating a sample of either **1(Pd)**·$2H_2O$ or **1(Pt)**·$2H_2O$ to 400 K and holding at this temperature for 1 h in the magnetometer to obtain **1(Pd)** or **1(Pt)**, respectively. Measurements were taken continuously in sweep mode at a scan rate of 2 K min^{-1} over the temperature range 400–50–350 K (Fig. 3(b)).
3) Resolvated samples of both Pd and Pt analogues were prepared by adding 1:1 EtOH:H_2O solvent into a sample holder containing a pre-prepared *in situ* dehydrated sample. Measurements were taken continuously in sweep mode at a scan rate of 2 K min^{-1} over the temperature range 250–50–250 K (Fig. S27).

Continuous cycling of hydrated and dehydrated Pd samples at 2 K min^{-1} (sweep mode) within the same temperature range (**1(Pd)**·$2H_2O$: 250–50–250 K; **1(Pd)**: 350–50–350 K) results in all cycles displaying the same SCO behaviour with only minor changes in the transition temperatures (Fig. S23). In contrast, the SCO behaviour of the hydrated and dehydrated phases of the Pt analogue diminishes with continuous cycling so is not reported here.

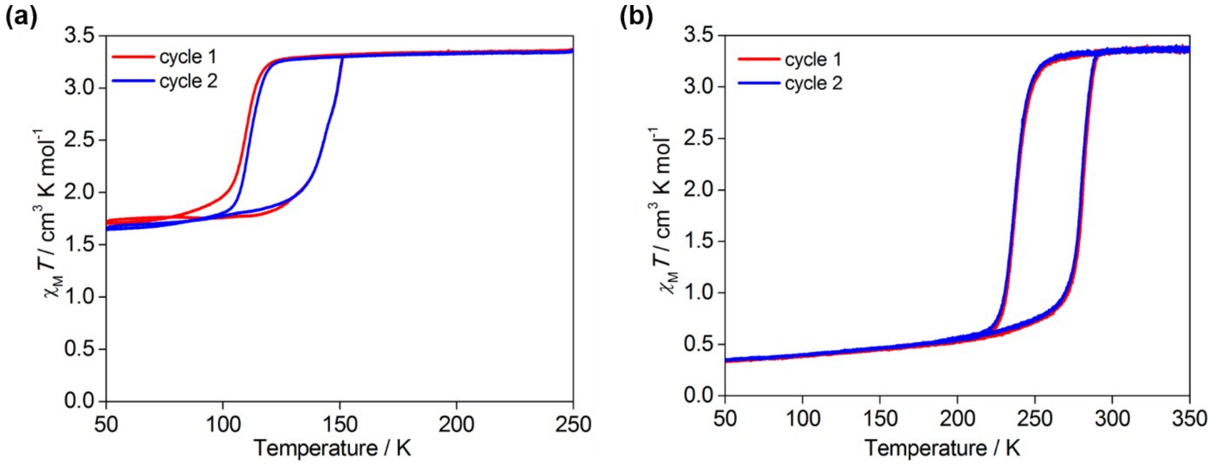

Fig. S23 Variable temperature magnetic susceptibility of (a) **1(Pd)**·$2H_2O$ (b) **1(Pd)** showing continuous cycling of the same sample at a scan rate of 2 K min^{-1}. Measurements were conducted in sweep mode.

The magnetic susceptibility of samples was also measured at different scan rates (1, 2 and 4 K min^{-1}) in sweep mode to determine kinetic effects (Fig. S24–26). For **1(Pd)**·$2H_2O$, **1(Pt)**·$2H_2O$, and **1(Pd)**, the hysteresis width is clearly scan rate dependent. For **1(Pd)**·$2H_2O$ and **1(Pt)**·$2H_2O$, $T_{½}\uparrow$ remains approximately constant for all rates but $T_{½}\downarrow$ decreases as the rate increases (Fig. S24(a) and S25(a)); whereas for **1(Pd)**, $T_{½}\downarrow$ remains approximately constant for all rates but $T_{½}\uparrow$ increases with increasing rate (Fig. S26(a)). In either case, faster scan rates result in a wider hysteresis loop. This expected trend is due to the small lag in the temperature of the sample compared to that of the magnetometer.[12, 13] Upon cooling at slower rates, the SCO sites have sufficient time to overcome the kinetic barrier and relax to the LS state, whereas faster rates overshoot the temperature before this occurs, resulting in a smaller $T_{½}\downarrow$ value, thus a wider hysteresis loop. For the hydrated phase, this is important when considering the rearrangement of the solvent guest molecules with cooling. Upon heating, however, reordering of the guest molecules isn't as important since the pore space increases, thus $T_{½}\uparrow$ remains approximately the same for all scan rates. It must be noted that the slight decrease in hysteresis width of **1(Pd)** compared to that reported in this manuscript is due to reduced crystallinity of the sample with repeated cycling (Fig. S26). In contrast, the SCO behaviour of **1(Pt)** diminishes considerably with cycling at different scan rates so is not reported here.

To extrapolate the 'true hysteresis width' corresponding to a 'zero scan rate', the $T_{½}$ values were plotted as a function of scan rate (Fig. S24(b), S25(b) and S26(b)). As only three scan rates were measured data were fitted using a linear function ($y = mx + b$). For **1(Pd)**·2H_2O, this results in 'zero scan rate' transition temperature values of $T_{½}$↓ = 122 K, $T_{½}$↑ = 147 K, ΔT = 25 K (Fig. S24(b)). For **1(Pt)**·2H_2O, this results in 'zero scan rate' transition temperature values of $T_{½}$↓ = 120 K, $T_{½}$↑ = 147 K, ΔT = 27 K (Fig. S25(b)). For **1(Pd)**, this results in 'zero scan rate' transition temperature values of $T_{½}$↓ = 234 K, $T_{½}$↑ = 278 K, ΔT = 44 K; this hysteresis width is still considerably large for SCO materials (Fig. S26(b)).

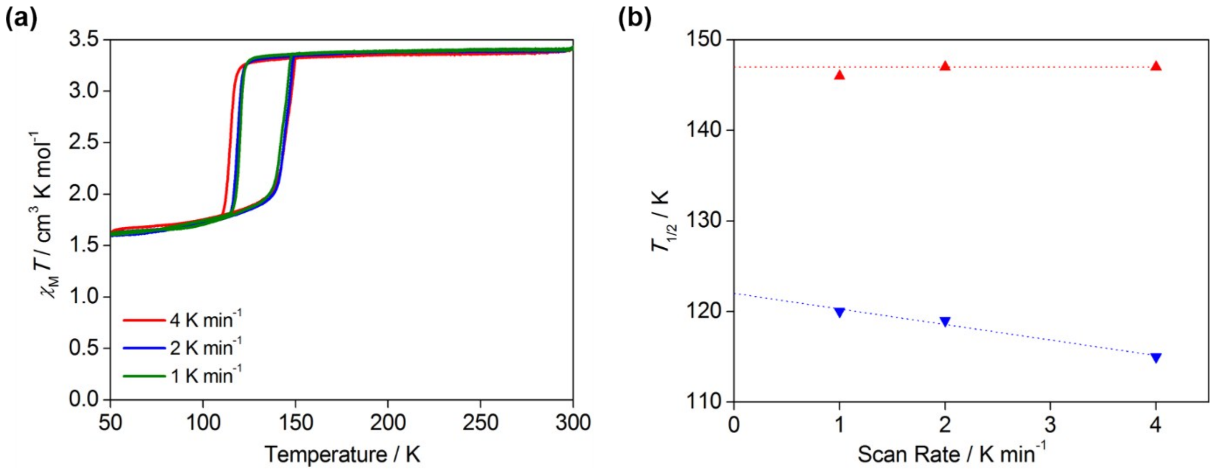

Fig. S24 (a) Variable temperature magnetic susceptibility of **1(Pd)**·2H_2O showing cycling of the sample at different scan rates (4, 2 and 1 K min^{-1}). Measurements were conducted in sweep mode. (b) The observed $T_{½}$↓ (▼) and $T_{½}$↑ (▲) values of **1(Pd)**·2H_2O as a function of scan rate used to extrapolate transition temperature values for a 'zero scan rate'. Data were fitted using a linear fit, and fits are shown as dotted lines.

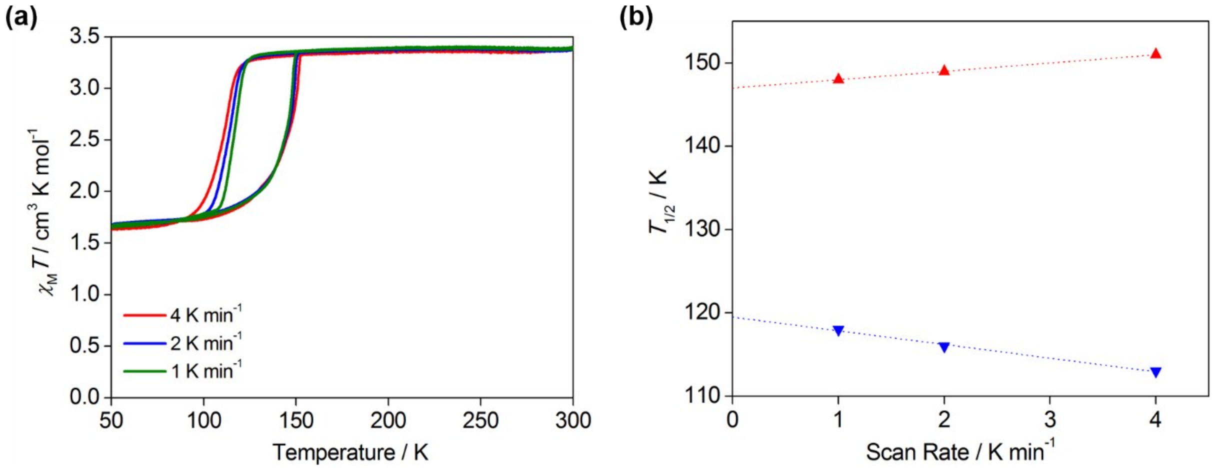

Fig. S25 (a) Variable temperature magnetic susceptibility of **1(Pt)**·$2H_2O$ showing cycling of the sample at different scan rates (4, 2 and 1 K min^{-1}). Measurements were conducted in sweep mode. (b) The observed $T_{½}$↓ (▼) and $T_{½}$↑ (▲) values of **1(Pt)**·$2H_2O$ as a function of scan rate used to extrapolate transition temperature values for a 'zero scan rate'. Data were fitted using a linear fit, and fits are shown as dotted lines.

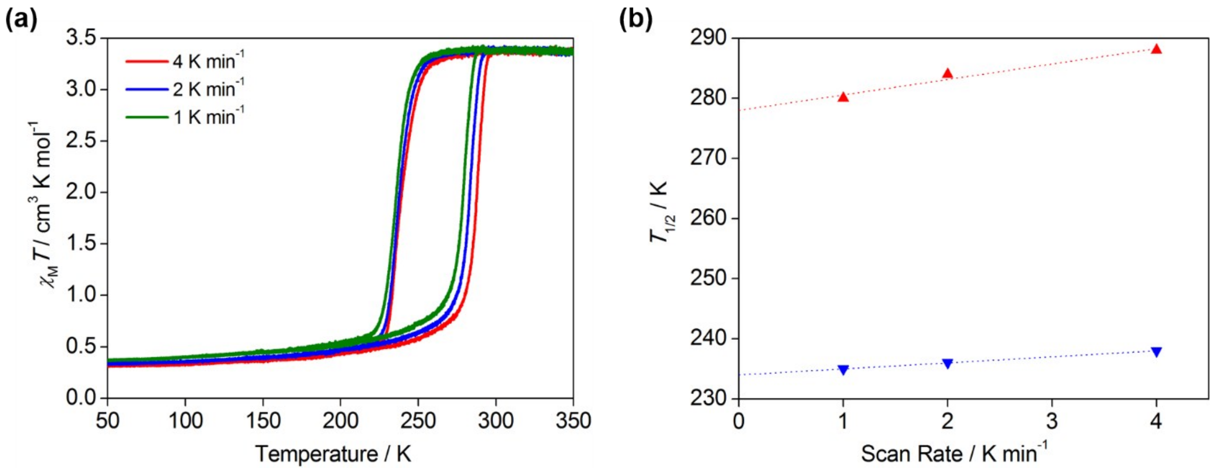

Fig. S26 (a) Variable temperature magnetic susceptibility of **1(Pd)** showing cycling of the sample at different scan rates (4, 2 and 1 K min^{-1}). Measurements were conducted in sweep mode. (b) The observed $T_{½}$↓ (▼) and $T_{½}$↑ (▲) values of **1(Pd)** as a function of scan rate used to extrapolate transition temperature values for a 'zero scan rate'. Data were fitted using a linear fit, and fits are shown as dotted lines.

Additionally, *in situ* dehydration of **1(Pd)**·2H_2O and **1(Pt)**·2H_2O followed by resolvation with a fresh 1:1 EtOH:H_2O solution results in the resolvated materials, **1(Pd)**^{R}·2H_2O and **1(Pt)**^{R}·2H_2O, respectively (Fig. S27). Magnetic susceptibility measurements on these resolvated samples (sweep mode, 2 K min^{-1}) affords incomplete, one-step, hysteretic SCO behaviour (**1(Pd)**^{R}·2H_2O: $T_{½}$↓ = 119 K, $T_{½}$↑ = 144 K, ΔT = 25 K; **1(Pt)**^{R}·2H_2O: $T_{½}$↓ = 121 K, $T_{½}$↑ = 149 K, ΔT = 28 K). Similarly to **1(Pd)**·2H_2O and **1(Pt)**·2H_2O, the magnetic susceptibility data of **1(Pd)**^{R}·2H_2O and **1(Pt)**^{R}·2H_2O correspond to a *ca.* 50% spin transition from a HS state to a 50:50% HS:LS state as a result of steric internal pressure effects. Thus, these resolvated materials have the same SCO behaviour as the as-made frameworks, **1(Pd)**·2H_2O and **1(Pt)**·2H_2O, but with slightly different transition temperatures.

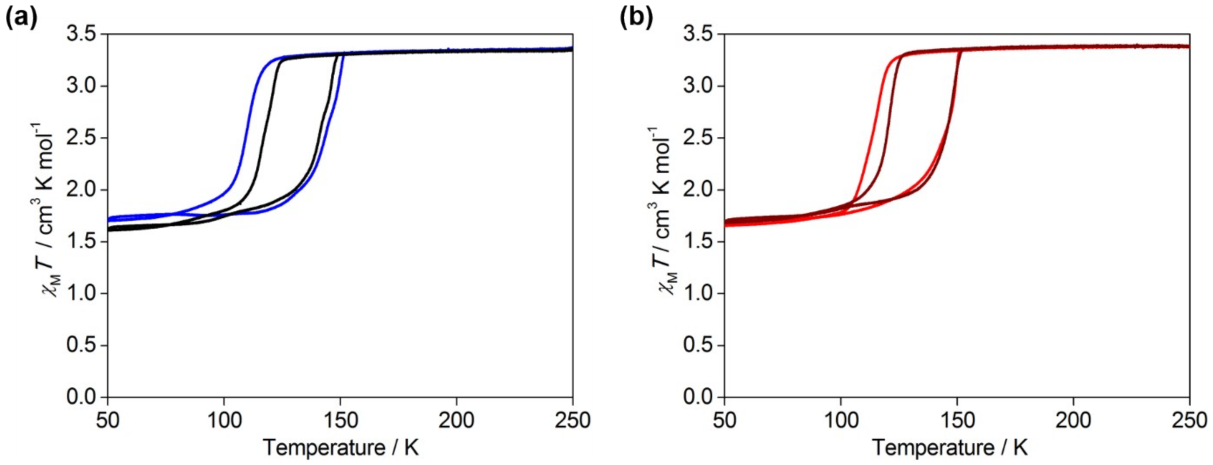

Fig. S27 Variable temperature magnetic susceptibility comparing the as-made and resolvated phases of (a) **1(Pd)**·2H_2O (blue) and **1(Pd)**^{R}·2H_2O (black), and (b) **1(Pt)**·2H_2O (red) and **1(Pt)**^{R}·2H_2O (maroon). Measurements were conducted in sweep mode at a scan rate of 2 K min^{-1}.

S5 – Variable Temperature Solid State UV-Visible Spectroscopy

UV-Vis spectra were collected over the range 5000–45000 cm^{-1} using a CARY 5000 spectrometer with a temperature probe. The set-up involved heating a sample of **1(Pd)**·2H_2O from room temperature to 100 °C at a rate of 1 °C min^{-1} under vacuum. The sample was enclosed in a shell containing two SiO_2 windows and one ZnS (observing) window. $BaSO_4$ was used as a background.

A band at 21740 cm^{-1} (460 nm) was observed for **1(Pd)**·2H_2O (Fig. 5, orange), which gradually shifts to 20540 cm^{-1} (487 nm) with dehydration to **1(Pd)** (Fig. 5, purple). This peak shift corresponds well with the colour change observed with dehydration from yellow to pink (Fig. 5 inset). This process is reversible whereby a dehydrated (pink) sample returns to the hydrated (yellow) phase when exposed to air, which is also evident from the UV-Vis spectra where the band returns to its original value of 21740 cm^{-1} (460 nm) with rehydration (in air).

S6 – Density Functional Theory Calculations

Standard density functional theory (DFT) calculations were carried out with Gaussian 09.[14] The geometries of the systems in Fig. S28 were extracted from the single crystal structure of **1(Pd)**·$2H_2O$ without modification.

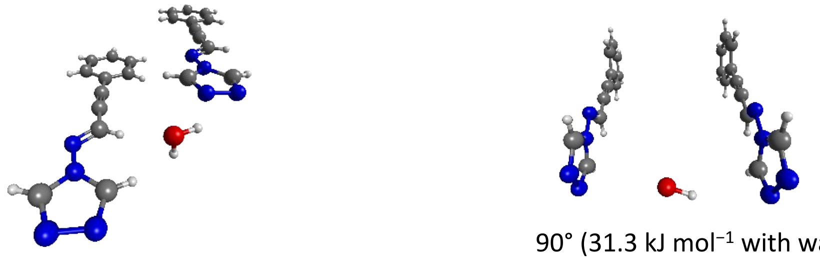

0° (0.0 kJ mol^{-1} with and without water)

90° (31.3 kJ mol^{-1} with water and 13.3 kJ mol^{-1} without water)

Fig. S28 Relative conformational energies of ligand dimers with and without water.

Relative conformational energies, shown above in parentheses (in kJ mol^{-1}), were calculated using the M06-2X[15] functional together with the 6-311+G(3df,2p) basis set. Time-dependent (TD) DFT calculations were carried out using the BMK procedure[16] with the 6-31G(d) basis set to compute the UV-Vis spectra, as in previous studies on similar systems.[17-21] A set of model systems, namely the monomeric ligand, and the dimeric forms in stacked and side-on arrangements, were used in the TD-DFT calculations. The lowest-energy major absorption peak for the monomer occurs at 24831 cm^{-1}, whereas for the stacked and side-on dimers, the peaks are at 23893 and 22251 cm^{-1}, respectively (Fig. S29).

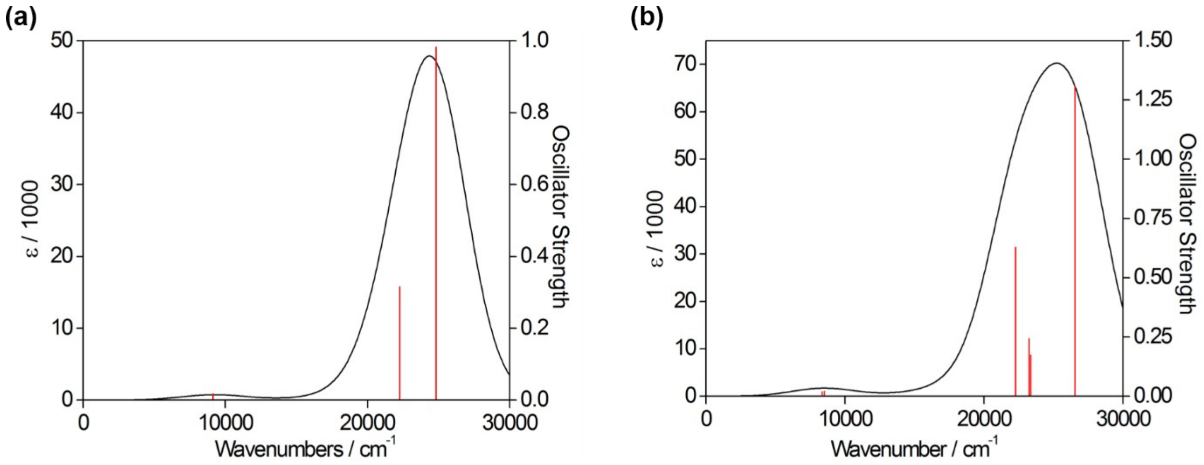

Fig. S29 Calculated gas phase UV-Vis spectra for (a) a monomer and (b) side-on dimers of proptrz ligands; simulated spectra (black), calculated excited states (red).

In order to inspect the SCO behaviour more directly, we have computed the LS and HS states for the model systems in Fig. S30. Thus, the combination of the two systems in Fig. S30(a) (with HCN ligands) and Fig. S30(b) (with CN^- ligands) represents the Fe core of the dehydrated system. To represent the hydrated system, we have included water molecules in our models in the preliminary studies (Fig. S28); however, we find that such inclusion, together with the use of a small truncated system, leads to excessive structural distortion. Thus, instead we use the protonated form to model the effect of water as a proton donor (Fig. S30(c)). A comparison of models Fig. S30(a) and Fig. S30(c) would then provide us with a qualitative picture of the effect of hydration. For this part of our DFT investigation, we used the TPSSh/def2-SVP method[22] for geometry optimisation and vibrational frequency calculation, as well as to obtain electronic energies. We note that this method and similar model systems have previously been successfully used to simulate SCO behaviour in related materials.[23]

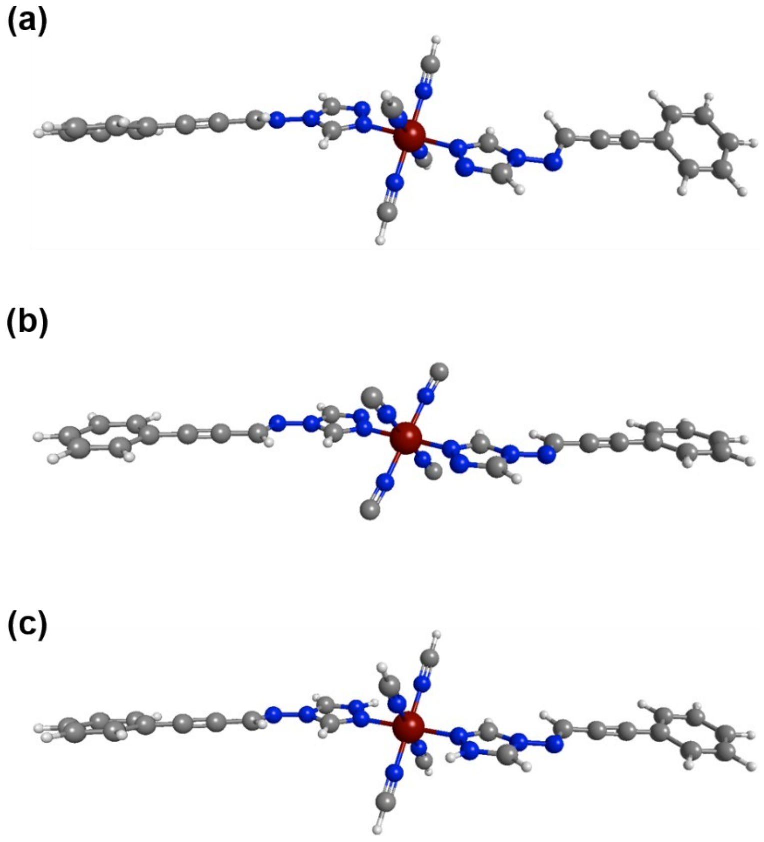

Fig. S30 Models used to represent the Fe core for simulating SCO behaviour of the dehydrated and hydrated materials. We have modelled these systems in both the HS and LS states, and shown here are the LS structures with (a) HCN ligands, (b) CN^- ligands, and (c) HCN and protonated proptrz ligands.

Fig. S31 shows plots of free energy differences between the HS and LS states [G(HS) − G(LS)] for the three models shown in Fig. S30. Thus, with the CN ligands capped with hydrogen (Fig. S30(a)), the relative energy is positive and the LS state is preferred over the entire temperature. On the other hand, the CN^- analogue (Fig. S30(b)) has the HS state as the preferred spin state. A linear combination of the free energy differences from the two models would thus yield a spin transition temperature within this range, which is what one might expect qualitatively. If we now compare models (a) and (c), it is apparent that protonation has a substantial effect of lowering the spin transition temperature. To approximately reproduce the experimental spin transition temperatures for the dehydrated phase, we used a 2:1 combination of models (a) and (b), which would yield a temperature of *ca.* 225 K. Likewise, a 2:1:1 combination of models (a), (b) and (c) would suggest a spin transition temperature of *ca.* 125 K for the hydrated material. The ratio between models (a) and (c) in this latter case are in line with our expectation that hydration would have a weaker effect than protonation.

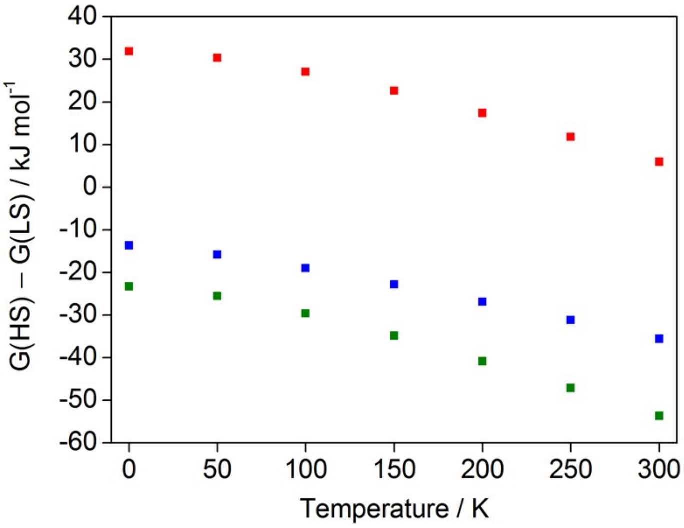

Fig. S31 Calculated relative free energies for models (a), (b) and (c) from Fig. S31 as a function of temperature.

S7 – Variable Temperature Raman Spectroscopy and DFT Calculations

Raman spectroscopy measurements were carried out using a Renishaw inVia Raman microscope equipped with a confocal set-up at 10× magnification. An excitation wavelength of 785 nm (1% laser power) was used for all measurements with a 30 s exposure time. Variable temperature measurements were carried out with a variable temperature stage attached to the instrument, with cooling achieved using liquid N_2, over the range 100–2400 cm^{-1} (3 accumulations). For the collection of each spectrum, the temperature was allowed to equilibrate for several minutes to ensure a stable temperature during measurement. Raman spectra were collected on **1(Pd)**·$2H_2O$ in 5 °C temperature intervals with gradual heating from 25 °C to 50 °C and then in 10 °C temperature intervals with gradual heating from 50 °C to 120 °C (using a 5 °C min^{-1} ramp rate). The temperature was held at 120 °C for 1 h to achieve *in situ* dehydration and another spectrum was collected, corresponding to the HS state of **1(Pd)**. The sample was cooled to −145 °C (using a 5 °C min^{-1} ramp rate) and a LS state spectrum of **1(Pd)** was collected.

Standard density functional theory (DFT) calculations were carried out using the Gaussian 09 program.[14] The $[Fe(proptrz)_2Pd(CN)_4]_n$ system was simulated using an Fe(II) complex extracted from the crystal structure of the dehydrated framework **1(Pd)** (i.e., the model obtained from Rietveld refinement) that was composed of two axial proptrz ligands and four equatorial cyanide ligands capped with hydrogen atoms. The complex was optimized using the PBE functional[24] and the 6-31G(d) basis set, with the spin multiplicity set to S = 1 for the LS structure and S = 5 for the HS structure. A solvation continuum was used to approximate the field effect of the bulk material, with the SMD[25] model used together with the parameters for benzene for modelling the aromatic-rich framework.[17, 26] All geometry optimisations were carried out using quadratically convergent SCF and an ultrafine grid with no symmetry imposed on the model. Harmonic frequency analysis was carried out after each geometry optimisation to ensure the structure corresponded to an equilibrium structure on the potential energy surface and to obtain the corresponding Raman spectrum.

With comparing the experimental Raman spectra of **1(Pd)** in the HS and LS states (Fig. S5(b), S32, Tables S6–S7), the alkyne (C≡C) stretch is shifted to lower frequencies in the HS state ($\nu_{C\equiv C}$ = 2195 cm^{-1}) compared to the LS state ($\nu_{C\equiv C}$ = 2200 cm^{-1}).

The cyanide (C≡N) stretching band occurs at ≈2200 cm^{-1} for both spin states as expected.[27, 28] The vibrations are shifted to higher frequencies compared to the frequency of free CN^- and discrete $K_2M(CN)_4$ compounds, which is expected for bridging cyanide linkers in an extended framework.[27-29] Also, this band occurs as a well-resolved doublet in the LS state indicating the symmetric and asymmetric stretching modes ($\nu_{C\equiv N}$ = 2163 and 2182 cm^{-1}, respectively), whereas there is only a single weak band present as a shoulder in the HS state spectrum corresponding to a symmetric C≡N

stretch ($\nu_{C≡N}$ = 2180 cm^{-1}). Since both the symmetric and asymmetric C≡N stretching bands are Raman active in the LS state but only the symmetric C≡N stretching band is Raman active in the HS state (the asymmetric C≡N stretching band is Raman inactive), this indicates a possible structural change between the two spins states. This complements the powder diffraction data in which the diffraction patterns are considerably different between the two spin states.

For both spin states, the bands in the region between 1600 and 990 cm^{-1} can be attributed to aromatic ring stretching and bending modes as outlined in Tables S6 and S7. Bands below 990 cm^{-1} could not be confidently assigned due to the low signal intensity; however, these may be attributed to weak metal–ligand vibrations as found in the Raman spectra of similar Hofmann-type frameworks.[27, 28]

In general, the peaks in the HS state spectrum have higher intensities than the peaks in the LS state spectrum. The reason for this may be due to a larger unit cell and longer Fe–N bond lengths in HS state, thus more space to allow more intense vibrations.

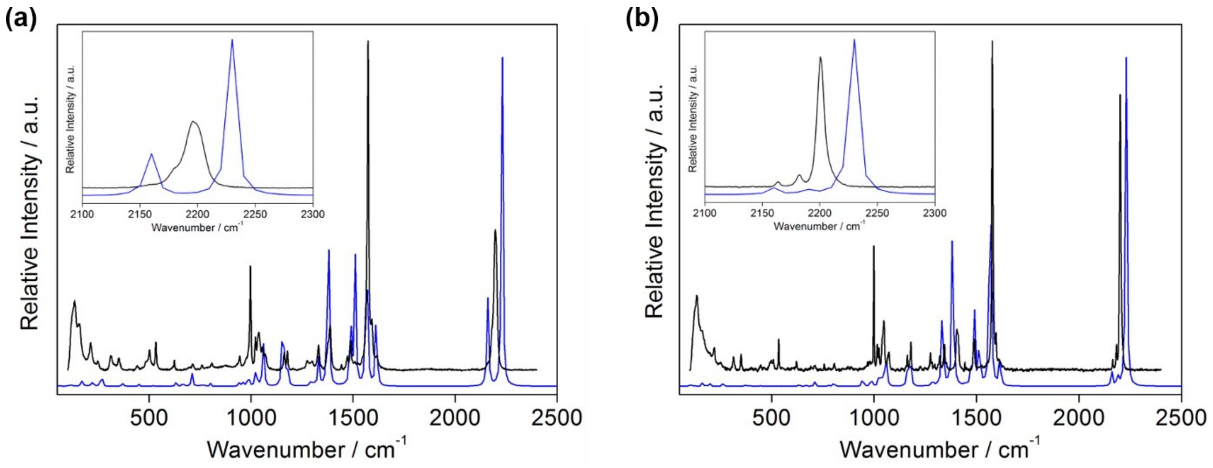

Fig. S32 Experimental (black) and calculated (blue) Raman spectra of **1(Pd)** in the (a) HS and (b) LS states.

Table S6 Experimental and corresponding calculated Raman vibrational modes of **1(Pd)** in the HS state assigned using DFT calculations. Vibrational modes are indicated by the arrows.

Experimental Vibrational Frequency / cm^{-1}	Calculated Vibrational Frequency / cm^{-1}	Vibrational Mode
2195	2229	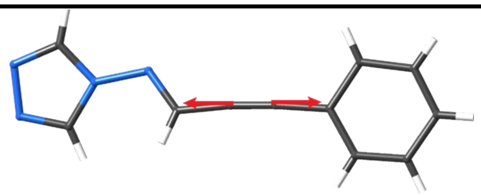
2180 (sh)	2161	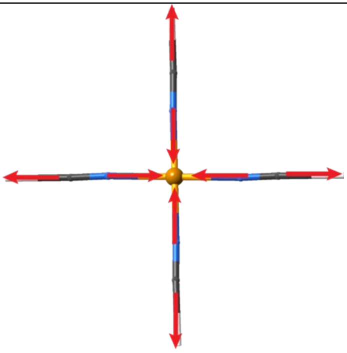
1591	1613	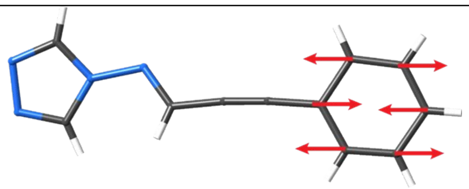
1573	1566	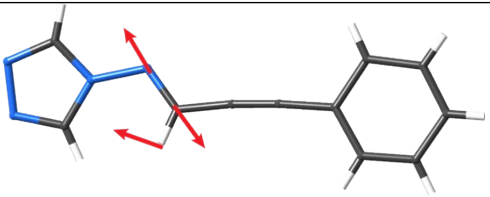
1488	1510	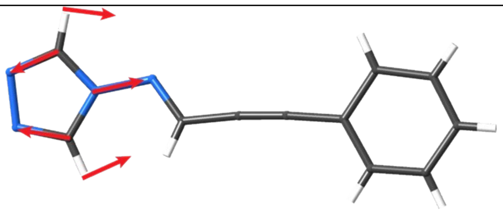
1471	1487	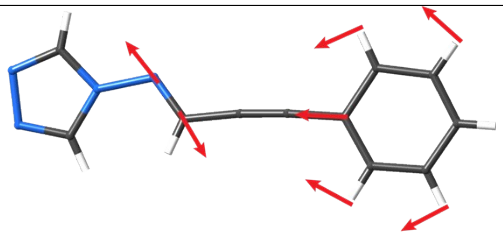

1387	1379	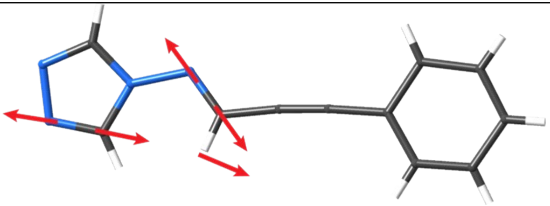
1329	1330	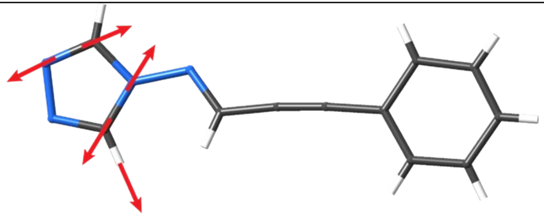
1177	1178	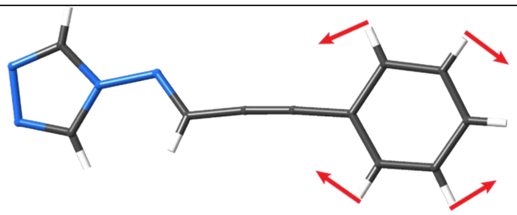
1160	1155	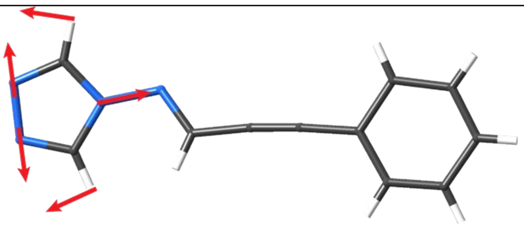
1037	1060	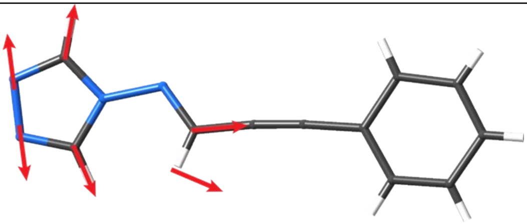
1020	1025	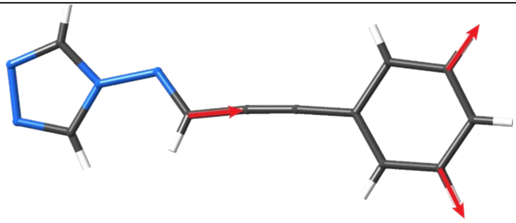
995	986	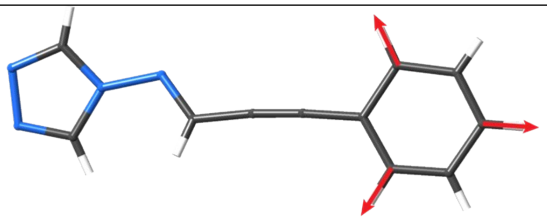

Table S7 Experimental and corresponding calculated Raman vibrational modes of **1(Pd)** in the LS state assigned using DFT calculations. Vibrational modes are indicated by the arrows.

Experimental Vibrational Frequency / cm^{-1}	Calculated Vibrational Frequency / cm^{-1}	Vibrational Mode
2200	2230	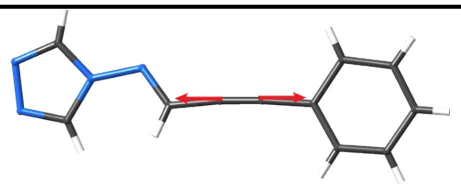
2182	2187	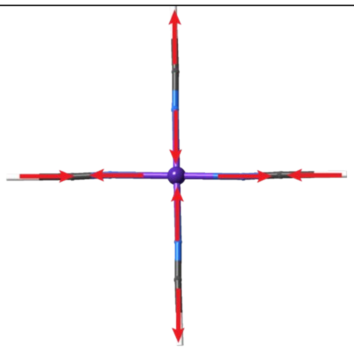
2163	2159	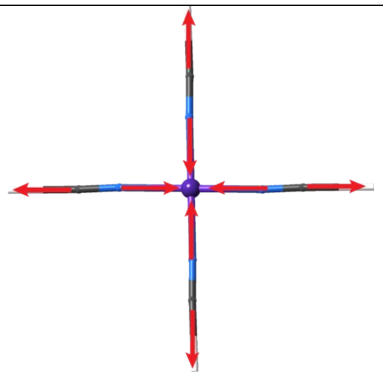
1594	1613	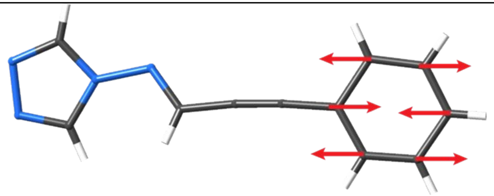
1577	1566	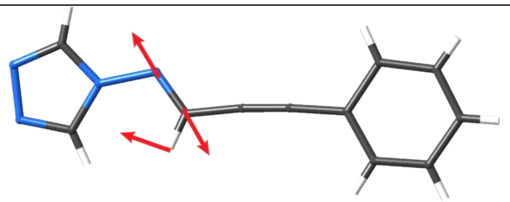
1489	1514	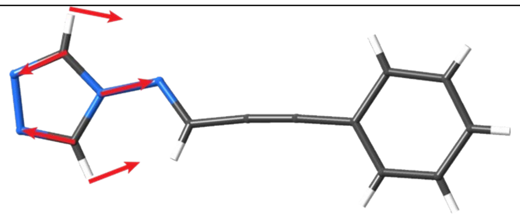

1483	1489	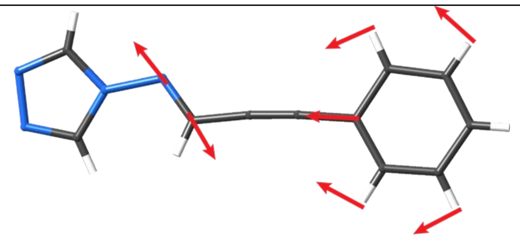
1403	1383	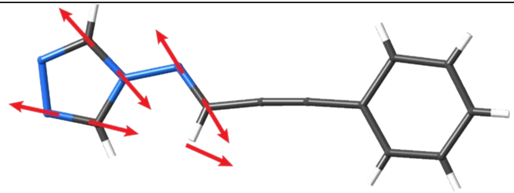
1342	1333	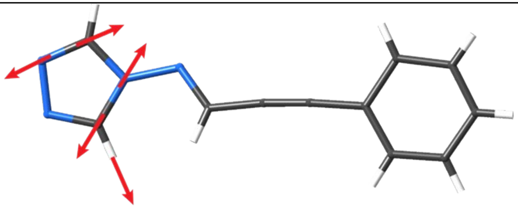
1179	1179	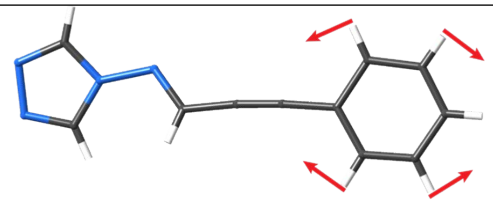
1162	1166	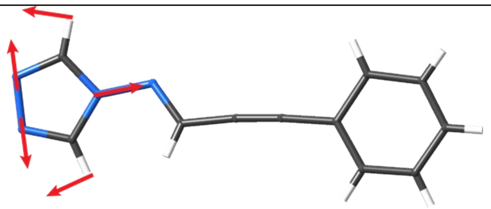
1072	1060	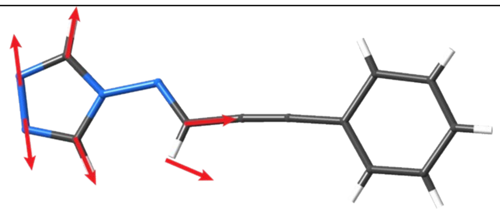
1047	1025	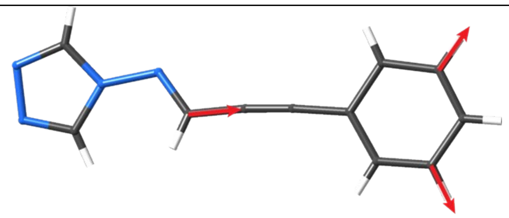
998	986	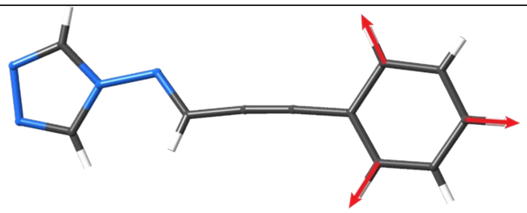

S8 – Thermogravimetric Analysis

Thermogravimetric experiments were performed on a TA Instruments Discovery TGA Thermogravimetric Analyser. Samples were prepared and loaded wet onto a platinum pan sample holder. Each sample was held at room temperature for 1 h prior to measurement to remove the surface solvent and then heated to 450 °C at a rate of 0.5 °C min^{-1} under a continuous stream of dry nitrogen gas (0.1 mL min^{-1}).

For **1(Pd)**·2H_2O, after removal of the surface solvent (not shown on the graph), guest molecules are gradually removed until 60 °C where there is a *ca.* 5% weight loss indicating a loss of 1.9 water molecules (Fig. S33, blue). The empty framework has a stable weight until 220 °C, above which decomposition occurs in two steps.

Similarly for **1(Pt)**·2H_2O, after removal of the surface solvent (not shown on the graph), guest molecules are gradually removed until 70 °C where there is a *ca.* 5% weight loss indicating a loss of 2.2 water molecules (Fig. S33, red). The empty framework has a stable weight until 230 °C, above which decomposition occurs in multiple steps.

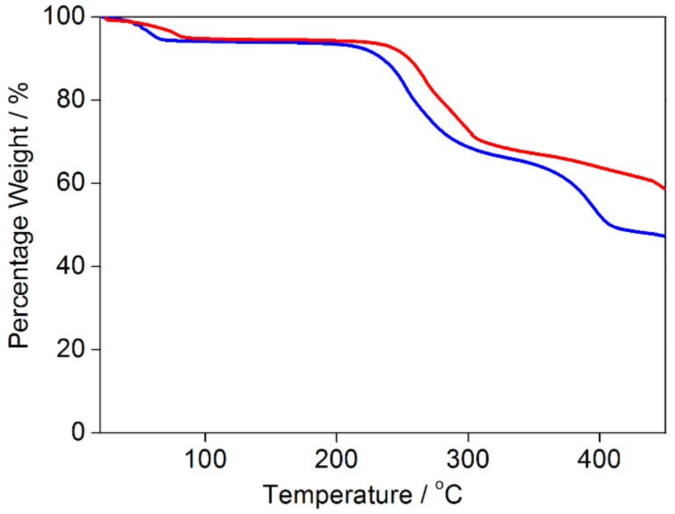

Fig. S33 Thermogravimetric analysis of the frameworks **1(Pd)**·2H_2O (blue) and **1(Pt)**·2H_2O (red) after a 1 h isothermal to remove the surface solvent.

S9 – References

1. CrysAlisPro v1.171.36.28, *Agilent Technologies XRD Products*, Oxfordshire, 2013.
2. G. M. Sheldrick, *SHELXS-97. Program for crystal structure solution*. University of Göttingen, Germany, 1997.
3. G. M. Sheldrick, *SHELXL-97. Program for crystal structure refinement*. University of Göttingen, Germany, 1997.
4. L. J. Barbour, *J. Supramol. Chem.*, 2001, **1**, 189–191.
5. A. L. Spek, *Acta Crystallogr.*, 1990, **A46**, 194–201.
6. A. P. Hammersley, S. O. Svebsson, M. Hanfland, A. N. Fitch and D. Hausermann, *High Pressure Res.*, 1996, **14**, 235–248.
7. A. P. Hammersley, *ESRF Internal Report*, 1997, **ESRF97HA02T**.
8. B. H. Toby and R. B. von Dreele, *J. Appl. Crystallogr.*, 2013, **46**, 544–549.
9. TOPAS v4.2, *Bruker ASX*, 2009.
10. N. F. Sciortino, K. R. Scherl-Gruenwald, G. Chastanet, G. J. Halder, K. W. Chapman, J. F. Létard and C. J. Kepert, *Angew. Chem. Int. Ed.*, 2012, **51**, 10154-10158.
11. M. J. Murphy, K. A. Zenere, F. Ragon, P. D. Southon, C. J. Kepert and S. M. Neville, *J. Am. Chem. Soc.*, 2017, **139**, 1330–1335.
12. R. G. Miller, J. L. Narayanaswamy, J. L. Tallon and S. Brooker, *New J. Chem.*, 2014, **38**, 1932–1941.
13. S. Brooker, *Chem. Soc. Rev.*, 2015, **44**, 2880–2892.
14. M. J. Frisch, G. W. Trucks, H. B. Schlegel, G. E. Scuseria, M. A. Robb, J. R. Cheeseman, G. Scalmani, V. Barone, B. Mennucci, G. A. Petersson, H. Nakatsuji, M. Caricato, X. Li, H. P. Hratchian, A. F. Izmaylov, J. Bloino, G. Zheng, J. L. Sonnenberg, M. Hada, M. Ehara, K. Toyota, R. Fukuda, J. Hasegawa, M. Ishida, T. Nakajima, Y. Honda, O. Kitao, H. Nakai, T. Vreven, J. J. A. Montgomery, J. E. Peralta, F. Ogliaro, M. Bearpark, J. J. Heyd, E. Brothers, K. N. Kudin, V. N. Staroverov, R. Kobayashi, J. Normand, K. Raghavachari, A. Rendell, J. C. Burant, S. S. Iyengar, J. Tomasi, M. Cossi, N. Rega, N. J. Millam, M. Klene, J. E. Knox, J. B. Cross, V. Bakken, C. Adamo, J. Jaramillo, R. Gomperts, R. E. Stratmann, O. Yazyev, A. J. Austin, R. Cammi, C. Pomelli, J. W. Ochterski, R. L. Martin, K. Morokuma, V. G. Zakrzewski, G. A. Voth, P. Salvador, J. J. Dannenberg, S. Dapprich, A. D. Daniels, Ö. Farkas, J. B. Foresman, J. V. Ortiz, J. Cioslowski and D. J. Fox, Wallingford CT, 2009.
15. Y. Zhao and D. G. Truhlar, *Theor. Chem. Acc.*, 2008, **120**, 215–241.
16. A. D. Boese and J. M. L. Martin, *J. Chem. Phys.*, 2004, **121**, 3405–3416.
17. C. F. Leong, B. Chan, T. B. Faust, P. Turner and D. M. D'Alessandro, *Inorg. Chem.*, 2013, **52**, 14246–14252.
18. C. F. Leong, B. Chan, T. B. Faust and D. M. D'Alessandro, *Chem. Sci.*, 2014, **5**, 4724–4728.
19. F. J. Rizzuto, T. B. Faust, B. Chan, C. Hua, D. M. D'Alessandro and C. J. Kepert, *Chem. Eur. J.*, 2014, **20**, 17597–17605.
20. F. J. Rizzuto, C. Hua, B. Chan, T. B. Faust, A. Rawal, C. F. Leong, J. M. Hook, C. J. Kepert and D. M. D'Alessandro, *Phys. Chem. Chem. Phys.*, 2015, **17**, 11252–11259.
21. C. Hua, B. Chan, A. Rawal, F. Tuna, D. Collison, J. M. Hook and D. M. D'Alessandro, *J. Mater. Chem. C*, 2016, **4**, 2535–2544.
22. V. N. Staroverov, G. E. Scuseria, J. Tao and J. P. Perdew, *J. Chem. Phys.*, 2003, **119**, 12129.
23. J. Cirera and F. Paesani, *Inorg. Chem.*, 2012, **51**, 8194–8201.
24. J. P. Perdew, K. Burke and M. Ernzerhof, *Phys. Rev. Lett.*, 1996, **77**, 3865–3868.
25. A. V. Marenich, C. J. Cramer and D. G. Truhlar, *J. Phys. Chem. B*, 2009, **113**, 6378–6396.
26. B. D. McCarthy, E. R. Hontz, S. R. Yost, T. Van Voorhis and M. Dincă, *J. Phys. Chem. Lett.*, 2013, **4**, 453–458.
27. G. Molnár, V. Niel, A. B. Gaspar, J. A. Real, A. Zwick, A. Bousseksou and J. J. McGarvey, *J. Phys. Chem. B*, 2002, **106**, 9701–9707.

28. G. Agustí, S. Cobo, A. B. Gaspar, G. Molnár, N. O. Moussa, P. Á. Szilágyi, V. Pálfi, C. Vieu, M. C. Muñoz, J. A. Real and A. Bousseksou, *Chem. Mater.*, 2008, **20**, 6721–6732.
29. K. Nakamoto, *Infrared and Raman Spectra of Inorganic and Coordination Compounds*, 6th edn., John Wiley & Sons, Inc., New Jersey, 2009.
